# Evolutionary insights and structural characterization guide the development of RAG1/RAG2-deficient swine models for immunological research

**DOI:** 10.3389/fimmu.2026.1757508

**Published:** 2026-03-25

**Authors:** Rui Zhang, Yunpeng Wu, Tianqi Sun, Tianqi Luan, Yanling Ren, Yilin Zhang, Lihao Lin, Teng Meng, Jin Wang, Yefeng Qiu

**Affiliations:** 1 Academy of Military Medical Sciences, Beijing, China; 2 Laboratory of Advanced Biotechnology, Beijing, China; 3 College of Life Science and Technology, Mudanjiang Normal University, Mudanjiang, China

**Keywords:** CRISPR/Cas9, evolutionary characteristics, immunodeficient pig models, protein docking, RAG1/RAG2, tertiary structural modeling

## Abstract

**Introduction:**

Defects in *RAG1/RAG2*-mediated V(D)J recombination cause severe combined immunodeficiency (SCID), a disorder characterized by the absence of mature T and B lymphocytes resulting from impaired antigen receptor gene rearrangement. Although evolutionary analyses demonstrate strong conservation of the catalytic core domains of RAG1/RAG2 across jawed vertebrates, structural divergence among species highlights the need for physiologically relevant large-animal models to evaluate human disease mechanisms. Pigs represent an advantageous model for human pathologies because of their close immunological and physiological similarities to humans.

**Methods:**

In this study, we performed evolutionary profiling, conservation analysis, tertiary structural modeling, and protein docking to characterize pig RAG1 and RAG2 and predict their functional roles in V(D)J recombination. These predictions were experimentally validated through the generation of *RAG1* and *RAG2* knockout pigs using CRISPR/Cas9-mediated genome editing.

**Results:**

Our findings define key structural features of pig RAG1 and RAG2 that are essential for V(D)J recombination and establish an integrated framework that combines comprehensive computational analyses with *in vivo* experimental validation.

**Discussion:**

This approach supports the development of pigs as physiologically relevant models of RAG deficiency and provides a foundation for future studies in pig immunology, genome editing, and translational research. Furthermore, these immunodeficient pigs permit long-term engraftment of human tissue xenografts and stem cell-derived teratomas, thereby enabling investigations of human immune reconstitution, cancer biology, and regenerative medicine. Collectively, this model offers a robust platform for immunotherapy development and for advancing studies in evolutionary and comparative immunology research.

## 1 Introduction

V(D)J recombination is the molecular mechanism responsible for assembling antigen receptor genes in jawed vertebrates that possess V(D)J-based adaptive immunity. By combinatorial rearrangement of variable (V), diversity (D), and joining (J) gene segments, the process generates extensive receptor diversity and thereby establishes the molecular foundation of adaptive immune responses. V(D)J recombination is catalyzed by the products of a tightly linked gene cluster, the recombination-activating genes RAG1 and RAG2 (1). Notably, no homologous gene pair with equivalent function have been identified in other species (2, 3).

Both RAG1 and RAG2 are essential for V(D)J recombination. The process is initiated when the RAG complex binds recombination signal sequences (RSSs) flanking antigen receptor gene segments and introduces site-specific double-stranded DNA breaks (1, 4). Although they function cooperatively, RAG1 and RAG2 play distinct roles during DNA cleavage. RAG1 contains the catalytic RNase H-like fold that is essential for DNA cleavage, as well as domains that directly contact RSSs, thereby conferring the enzymatic activity of the RAG complex (1, 4–6). In contrast, RAG2 primarily serves a regulatory role: it interacts with RAG1 to enhance DNA binding and cleavage efficiency. Additionally, RAG2 contains a plant homeodomain (PHD) finger that specifically recognizes histone H3 trimethylated at lysine 4 (H3K4me3), promoting recruitment of the RAG complex to transcriptionally active chromatin (1, 4).

Since the discovery of *RAG1* and *RAG2* genes, multiple crystal structures of RAG1–RAG2 complexes have been resolved. In mice, RAG1 (1,040 residues) and RAG2 (527 residues) function cooperatively in all known functions of the recombinase complex. Structural and functional studies have delineated the catalytic core, regulatory regions, active-site residues, DNA-binding domains, two zinc-binding motifs, and key elements of the RAG1-RAG2 interaction interface (5). The catalytic core of mouse RAG1 (residues 384–1008) comprises seven structural modules. The N-terminal nonamer-binding domain (NBD, residues 391-459) forms a domain-swapped dimer, followed by the dimerization and DNA binding domain (DDBD; residues 459-515), which is connected to the NBD by a flexible linker. Completion of the DDBD structure requires folding back of the last helix of the three-helix C-terminal domain (CTD; residues 962-1008). Downstream of the DDBD lie the extended pre-RNase H domain (preR; residues 515-588), the catalytic RNase H domain (RNH; residues 588-719), and the ZnC2 domain (residues 719-791), which project toward RAG2. The highly helical ZnH2 domain (residues 791-962) contributes substantially to the three-dimensional (3D) architecture of RAG1 and repositions E962 within the catalytic center (5, 7–9). Three conserved carboxylate residues including D600, D708, and E962, are essential for catalytic activity. The core region of the mouse RAG1 (residues 1-352) adopts a six-bladed β-propeller structure composed of tandem kelch repeats and mediates interactions with chromatin. In contrast, the noncore C-terminal region contains a plant homeodomain (PHD) finger that is critical for epigenetic regulation (7, 8). Biochemical and functional studies have demonstrated that truncated ‘core’ RAG proteins (RAG1 residues 384–1008 and RAG2 residues 1-387) retain targeted DNA cleavage activity *in vitro* and support recombination in cells, albeit with reduced regulatory control (5, 10).

Null mutations in *RAG1* or *RAG2* disrupt V(D)J recombination in mammals and result in severe combined immunodeficiency (SCID), characterized by thymic hypoplasia, splenic abnormalities, and loss of lymphocyte diversity (11–16). Numerous pathogenic variants in human RAG1 and RAG2 have been identified, leading to SCID syndrome or the milder Omenn syndrome (5). Rodent models, particularly *RAG1-* and *RAG2-* deficient mice, have been instrumental in elucidating SCID pathogenesis (17–19). Immunodeficient mice are also widely used for reconstitution of the human hematopoietic system through transplantation of human hematopoietic stem cells (20). Additional models, including *RAG1*-deficient rats, *RAG1/2*-deficient rabbits, *RAG1*-deficient chickens and *RAG1/IL2RG*-deficient monkeys, have also been developed (21–26). Despite their utility, these models have significant limitations. Rodents and midsize animals such as rabbits often inadequately mimic human genetic and physiological conditions. In particular, they cannot reproduce human organ size, immune microenvironment dynamics, or inflammatory responses, thereby constraining translational applicability (27–29). Their small body size and relatively short lifespan further limit surgical manipulations, clinical procedures, and long-term monitoring following tissue or cell transplantation (30). As a result, preclinical drug screening and cell therapy studies conducted in SCID mice often show limited predictive value for human outcomes. Although nonhuman primates (NHPs) offer closer genetic and physiological similarity to humans, their use is limited by high costs, limited availability, and significant ethical considerations.

Pigs share significant genetic, anatomical, and physiological similarities with humans, including comparable organ size and lifespan (30, 31). Importantly, their immune system exhibits a high degree of evolutionary conservation, particularly in lymphoid development and the mechanism governing V(D)J recombination. These features make pigs highly suitable models for biomedical research, especially in tissue engineering and the generation of humanized tissues or organs for xenotransplantation (11, 12, 32–34). Gene editing-mediated disruption of *RAG1*- or *RAG2*-knockout pigs reliably reproduces key features of human SCID, including thymic atrophy, absence of mature T and B lymphocytes, and loss of V(D)J recombination (11, 12, 30, 35). As a result, SCID pig models provide a powerful platform for long-term investigations of xenotransplantation, immune responses, stem cell biology, and cancer progression under clinically relevant conditions. They also enable the development of strategies to evaluate stem cell therapy safety and to assess surgical and radiotherapeutic interventions in transplanted tumors. Collectively, SCID pigs represent a valuable preclinical system for translational research (35–38). However, significant knowledge gaps remain in accurately modeling the spectrum of human immunodeficiency disorders.

Although the core mechanism of RAG1 and RAG2 function appear to be evolutionarily conserved, the evolutionary trajectories and species-specific divergence of RAG genes in pigs remain poorly defined (39). In particular, systemic analyses of structural domain conservation, especially within catalytic core regions, and noncore regulatory domains implicated in immune dysregulation, are lacking. To address these gaps and to evaluate the suitability of SCID pig models for studying diverse forms of human immunodeficiency, we performed comprehensive evolutionary and structural characterizations of pig RAG1 and RAG2. Based on these findings, we generated SCID pigs through targeted disruption of *RAG1* and *RAG2* using CRISPR/Cas9-mediated genome editing combined with somatic cell nuclear transfer (SCNT), followed by detailed phenotypic analysis. This work provides guidance for the development of robust SCID pig models and establishes a foundation for their application in studies of adaptive immune deficiency, assessment of immune reconstitution therapies, and modeling of human SCID pathogenesis.

## 2 Materials and methods

### 2.1 Ethics statement

All animal procedures were reviewed and approved by the Institutional Animal Care and Use Committee (IACUC) of the Academy of Military Medical Sciences (Approval No. IACUC-DWZX-2024-013). All experiments were conducted in strict accordance with the Guide for the Care and Use of Laboratory Animals issued by the National Institutes of Health (NIH).

### 2.2 Phylogenetic analysis of *RAG1* and *RAG2*

Protein sequences of vertebrate RAG1 and RAG2 were obtained from the NCBI database. Detailed species information and number of sequences analyzed are listed in **Supplementary Table 1**. Pseudogenes were excluded from all analyses. Multiple sequence alignments of full-length vertebrate RAG1 and RAG2 proteins was generated using MUSCLE implemented in MEGA7. To improve alignment quality and maximize informative regions, poorly aligned positions and gap-rich columns were removed using Gblocks and trimAI within the PhyloSuite platform.

Maximum-likelihood phylogenetic trees were reconstructed using PhyML v3.0. Optimal substitution models were selected using the Smart Model Selection (SMS) algorithm implemented on the PhyML web server (40–42). Branch support values were estimated using approximate likelihood ratio tests (aLRT) with SH-like interpretation (43). Specifically, the RAG tree was constructed under the Q.plant+G+I model, the RAG1 tree was constructed under the Q.bird+G+I model, and the RAG2 tree employed the Q.plant+G+I+F model. All analyses employed four discrete rate categories. Final tree visualization, annotation of topological features, and graphical refinement were performed using Evolview v3.0 to enhance interpretability of evolutionary relationships and nodal support values (44).

### 2.3 Residue conservation analysis

Evolutionary conservation of RAG1 and RAG2 residues was assessed using the ConSurf server (45). Structural modeling was performed using a hierarchical approach. Briefly, homologous templates were identified in the Protein Data Bank (PDB) using HHPred (v3.0), which applies hidden Markov model-based sequence alignment. 3D models were subsequently generated using MODELLER (v10.4). The X-ray crystal structures 6DBJ (Chain A) and 6V0V (Chain B) were used as primary templates for RAG1 and RAG2, respectively. Conservation scores ranging from 1 to 9 were mapped onto the predicted structures, where grade 1 represents highly variable (rapidly evolving) residues and grade 9 denotes highly conserved (slowly evolving) positions. Final visualizations were generated by mapping sequence conservation profiles of pig RAG1 and RAG2 onto both sequence conservation patterns and the predicted structures using surface rendering in PyMOL.

### 2.4 Structural modeling

The tertiary structures of pig RAG1 and RAG2 were independently predicted using Phyre2 (v2.2) (46, 47), which performs profile-based alignment of hidden Markov models using HHBlits. Structural refinement, topological annotation, and molecular visualization were subsequently conducted in PyMol (48).

### 2.5 Protein–protein docking analysis

To investigate the interaction mechanisms between pig RAG1 and RAG2, systematic molecular docking analyses were performed. The predicted 3D structures of pig RAG1 and RAG2 were submitted to the ZDOCK server (49) as receptor and ligand, respectively. ZDOCK performs an exhaustive search of potential binding conformations in both translational and rotational space and ranks docking poses using an energy-based scoring function. To further characterize the interaction interface, we performed a detailed analysis of the pig-RAG1–2 complex using the PDBePISA interactive tool. In addition, we used HDOCK (50), which integrates template-based modeling with ab initio free docking algorithms to predict the structure of the RAG1-RAG2 heterotetramer. The structural refinement and molecular visualization were performed in PyMol and Jmol (48, 51).

### 2.6 Generation of *RAG1/RAG2*-knockout pigs via the CRISPR/Cas9 system

*RAG1* and *RAG2* double-knockout (DKO) pigs were generated using CRISPR/Cas9-mediated genome editing combined with somatic cell nuclear transfer (SCNT). The single-guide RNA (sgRNA) targeting *RAG1* was designed within the importin-binding domain (residues 11–291 in pigs), which mediates interaction with importin-alpha and is located upstream of the RING/Zinc finger (RING) domain (52). The sgRNA targeting *RAG2* was designed within the β-propeller domain. The sgRNA sequences are RAG1-CATGTGAGGTTTACTCCCCAAGG and RAG2-ATAAGGGTTGATCTCCCCCTGGG, and Cas9 expression vectors carrying blue or red fluorescent markers were constructed using primer sequences listed in **Supplementary Table 4**. The sgRNAs and Cas9 plasmids were transfected into pig fetal fibroblasts (PFFs). Forty-eight hours post transfection, successfully transfected cells were enriched by fluorescence-activated cell sorting (FACS). Genomic DNA isolated from clones was subjected to PCR amplification (primers listed in **Supplementary Table 4**) followed by Sanger sequencing to confirm targeted mutations. Verified *RAG*1/*RAG2* DKO cell lines were subsequently used as donor cells for SCNT. Founder animals were genotyped after birth by PCR amplification and Sanger sequencing of the targeted regions within the pig *RAG1* and *RAG2* genes. Comprehensive phenotypic analysis was then performed to assess the biological consequences of *RAG1*/*RAG2* double knockout. To assess V(D)J recombination, genomic DNA were extracted from whole blood of mutant and control pigs. T-cell receptor β chain (TCRβ) gene rearrangement was analyzed using the D1J1-F and D1J1 primer pair. Immunoglobulin heavy chain (IgH) recombination was detected using the FR1 and JH primers to amplify rearranged alleles, while D4-F and J3-R primers were used to detect the germline configuration as an internal control (**Supplementary Table 4**) (12). Additionally, thymic and splenic anatomy was examined. The proportions of CD4^+^ T cells, CD8^+^ T cells and γδ T cells in peripheral blood of *RAG1*/*RAG2* DKO piglet were determined by flow cytometry using the following antibodies: #MCA1222A647 (AbD Serotec); #55958 (5BD), #559584 (BD), #561482 (BD), and #561477 (BD).

## 3 Results

### 3.1 Phylogenetic consistencies of RAG1 and RAG2 topologies suggest functionally coupled co-evolution

**Figure 1:**
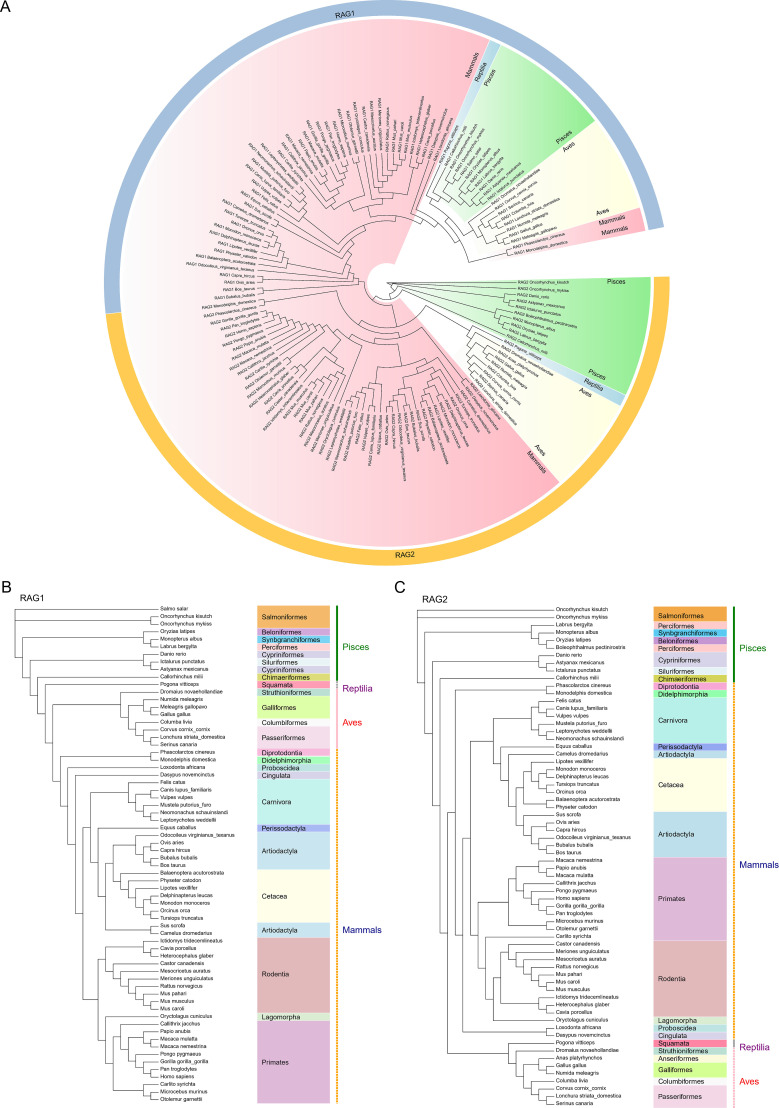
Phylogenetic analysis of pig and vertebrate RAG1 and RAG2. **(A)** Large-scale maximum-likelihood phylogeny of vertebrate RAG proteins constructed from full-length RAG sequences. Major taxonomic clades are color-coded, and selected branches are collapsed to simplify visualization. **(B)** Expanded view of the pig RAG1 clade. Pig RAG1 clusters most closely with camel RAG1, indicating strong orthology. **(C)** Expanded view of the pig RAG2 clade. Pig RAG2 forms a well-supported orthologous group with sheep and goat RAG2.

*RAG1* and *RAG2* are encoded by a tightly linked gene cluster. To investigate their evolutionary history, we retrieved 66 full-length vertebrate RAG1 sequences and 66 RAG2 sequences and constructed maximum-likelihood (ML) phylogenetic trees (**Figure 1**). Phylogenetic analysis demonstrated that mammalian RAG1 proteins are highly conserved. The most evolutionarily distant RAG1 sequences in our dataset, derived from *Salmo salar* and *Otolemur garnettii*, shared 58.68% overall sequence identity, consistent with previous reports (53, 54). Analysis of RAG2 revealed a similarly high degree of overall conservation, although slightly lower than that of RAG1. The most divergent RAG2 sequences, *Oncorhynchus kisutch* and *Serinus canaria*, shared 48.86% overall sequence identity. This greater divergence may reflect distinct evolutionary origins and functional constraints. Genomic evidence suggests that the RAG1 core region evolved from a *Transib* transposase, whereas RAG2 likely acquired novel functions through functional integration with RAG1 during vertebrate evolution. Importantly, the phylogenetic topologies of RAG1 and RAG2 were highly congruent, supporting a functionally coupled co-evolutionary relationship between these two genes, as previously proposed (3, 16, 54). Within mammals, pig RAG1 clustered most closely with camel RAG1, while pig RAG2 formed a well-supported orthologous clade with sheep and goat RAG2.

### 3.2 Strong evolutionary conservation of the RAG1 core region and the β-propeller and PHD domains of RAG2

**Figure 2:**
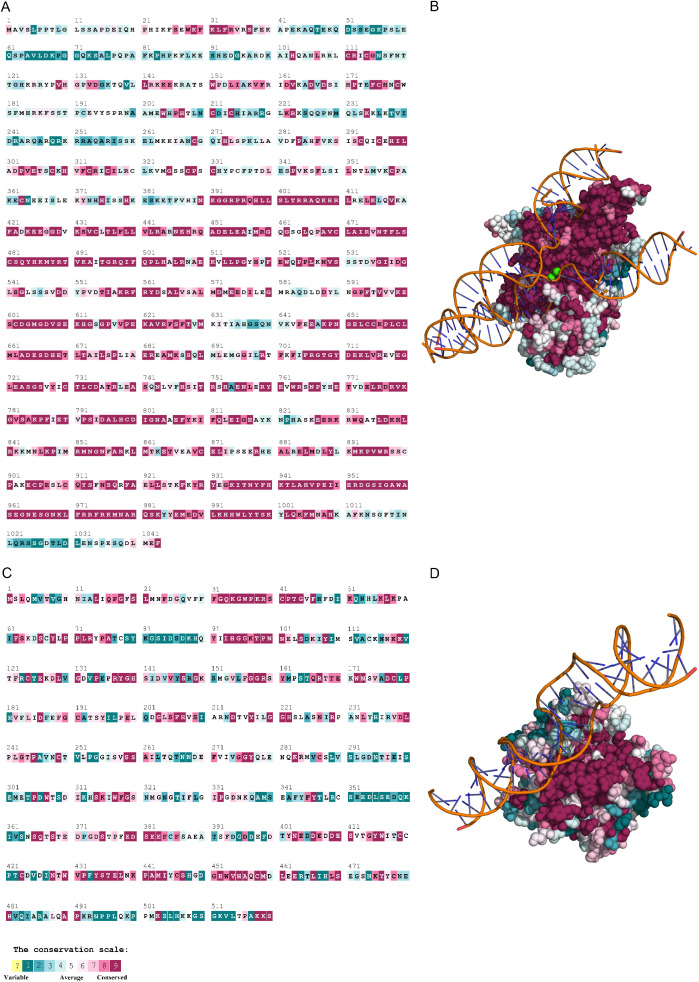
Evolutionary conservation of amino acid residues in pig RAG1 and RAG2. **(A, C)** Linear mapping of conservation grades along the primary sequences of pig RAG1 **(A)** and RAG2 **(C)**. **(B, D)** Structural mapping of the conservation grades onto the predicted 3D models of pig RAG1 **(B)** and RAG2 **(D)**. Conservation scores range from 1 (most variable; turquoise) to 9 (most conserved; maroon). Residues with low-confidence conservation assignments are shown in light yellow. Conservation analysis was performed using pig RAG1 and RAG2 sequences to define the nine-grade color scale.

Based on the phylogenetic relationships among homologous sequences, residue-specific evolutionary conservation scores for RAG1 and RAG2 were calculated and mapped using ConSurf (**Figure 2**). The analysis revealed differential conservation patterns across functional domains.

Pig RAG1 is a 1043-amino acid protein, similar in length to human RAG1, and contains a catalytically active core region (residues 387–1011). This evolutionarily conserved core encompasses two critical structural elements: the nonamer-binding domain (NBD), responsible for DNA recognition, and the catalytic triad (D603, D711, and E965), which is essential for enzymatic activity. In addition, the C3H4-type zinc finger (RING-finger; residues 293-331) and adjacent zinc-binding domains (residues 354–383) are highly conserved. Pig RAG2 consists of 527 amino acids and contains a conserved core region that forms a β-propeller domain (residues 1–352 in humans, pigs, and mice), as well as a conserved noncore C-terminal plant homeodomain (PHD) finger. Key hydrophobic and catalytic residues within these functional domains—particularly those in the RAG1 core—are exceptionally conserved across vertebrates, especially among closely related taxa, indicating strong purifying selection and slow evolutionary rates. In contrast, residues outside of these essential domains exhibit greater variability and are inferred to evolve more rapidly. These conservation patterns demonstrate the functional importance of specific structural regions in RAG1 and RAG2. **Figure 2** illustrates the distribution of conserved residues in both the primary sequence and 3D structures of pig RAG1 and RAG2, highlighting the critical roles of evolutionary constrained positions in maintaining recombinase activity.

### 3.3 Structural analysis of pig RAG1 and RAG2

**Figure 3:**
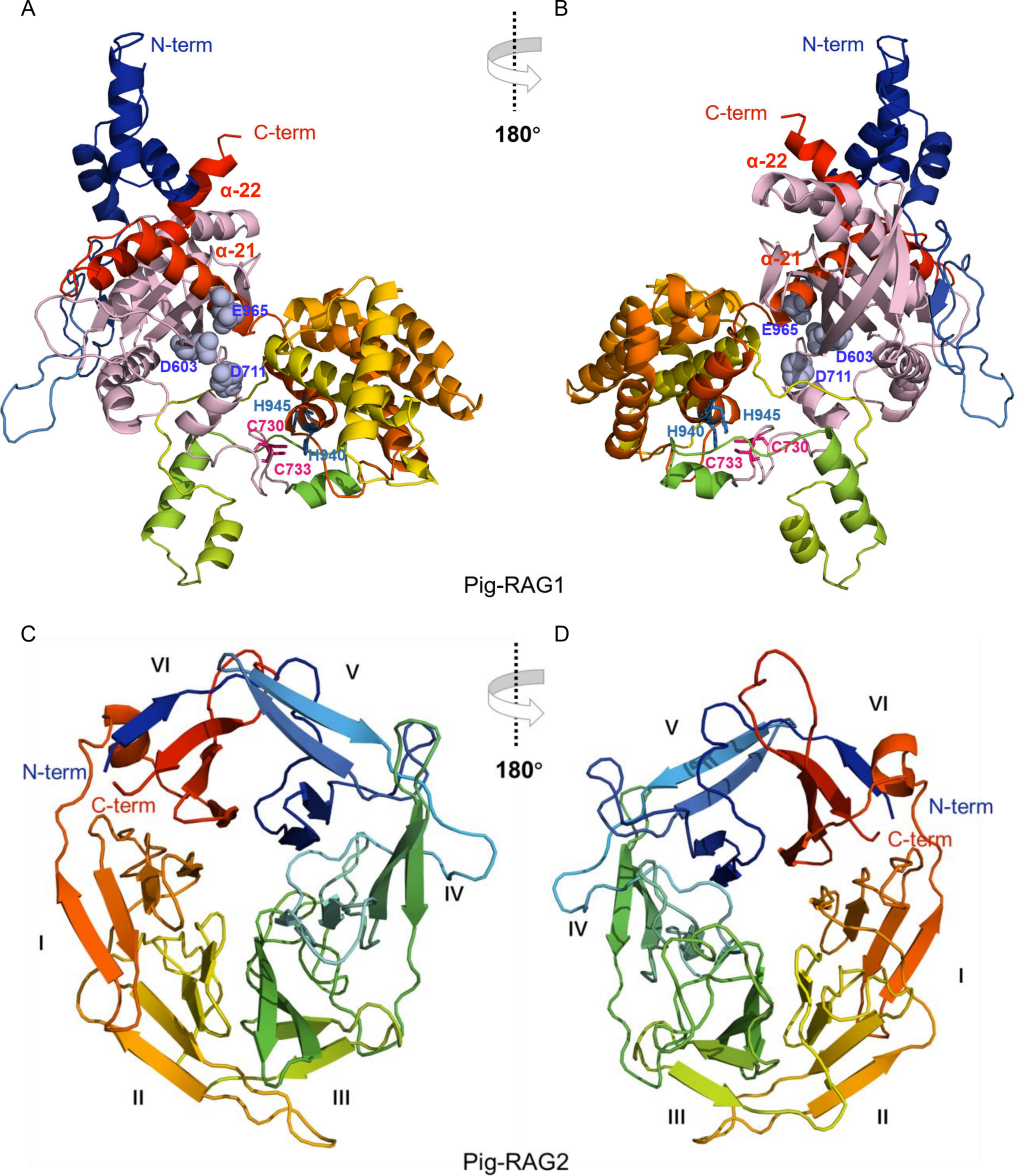
Structural models of pig RAG1 and RAG2. **(A)** Predicted 3D structure of pig RAG1. **(B)** Rotated view (180°) of the pig RAG1. **(C)** Predicted 3D structure of pig RAG2. **(D)** Rotated view (180°) of the pig RAG2. The catalytic triad (D603, D711, and E965 in pigs) is marked with spheres in light blue. The first zinc-binding region (C730, C733 in pigs) is marked with hot-pink sticks, and the second zinc-binding region (H940, H945 in pigs) is marked with sky‑blue sticks. Two-helix bundles (α21 and α22) are marked with the corresponding color in the 3D structure.

Given the high sequence homology between pig RAG1/RAG2 and their vertebrate counterparts, 3D structural models were generated using the Phyre2 server (intensive mode) (**Figure 3**).

The pig RAG1 molecular structure was modelled using Chain A of the *Danio rerio*-RAG complex (PDB ID: 6DBJ) as the template. The model was generated with 100% confidence, approximately 77% sequence identity, and covered 53% of pig RAG1 (548 residues), primarily within the catalytic core region (**Figures 3A, B**). The pig RAG2 molecular structure was modelled using Chain D of *Danio rerio*-RAG complex (PDB ID: 6DBJ) as the template, and generated with 100% confidence, approximately 51% sequence identity, and covered 66% of pig RAG2 (350 residues) in its β-propeller domain (**Figures 3C, D**). The predicted 3D structure of the pig RAG1 (**Figure 3A**) core region indicated that it is composed of 22 α-helices and 8 β-strands. The catalytic triad (D603, D711, and E965 in pigs; shown as light-blue spheres) clusters within the catalytic domain (residues 555–735; highlighted in light-pink). Within the C-terminal domain (CTD), a two-helix bundle (α21 and α22) is present, and the first helix (α21) contributes E965 (**Figures 3A, B**) to the active site. This configuration is consistent with proper positioning of the catalytic center and facilitates and interaction with RAG2. Two residues in the first zinc-binding region (C730 and C733; shown as hot-pink sticks) and two in the second region (H940 and H945; shown as sky-blue spheres) converge spatially despite being in the primary sequence, forming a unified zinc-binding site (5, 55). This zin-binding site is positioned adjacent to the catalytic center and interfaces with the RAG2-binding (ZnC2), DDBD, and ZnH2 domains, thereby structurally integrating catalytic and protein-protein interaction modules.

The predicted RAG2 core consists of one α-helix and 24 β-strands arranged into a stable six-bladed β-propeller formed by kelch repeats, with each β-propeller composed of four β-strands. The N-terminal β-strand integrates into the sixth blade, structurally bridging the first and last kelch repeats. The distal face of the β-propeller contains extended loops, a characteristic feature of the fold (**Figure 3C**). Notably, loops located between adjacent blades and within individual blades interact extensively with the pre-RNase H (preR), RNase H (RNH), and ZnC2 domains of RAG1. The RAG2 core structure mediates chromatin-associated functions, while the noncore C-terminal region contains a PHD finger domain (residues 414–491 in pigs) implicated in epigenetic regulation (9).

### 3.4 Pig RAG1-RAG2 form functional heterodimers and heterotetramers

**Figure 4:**
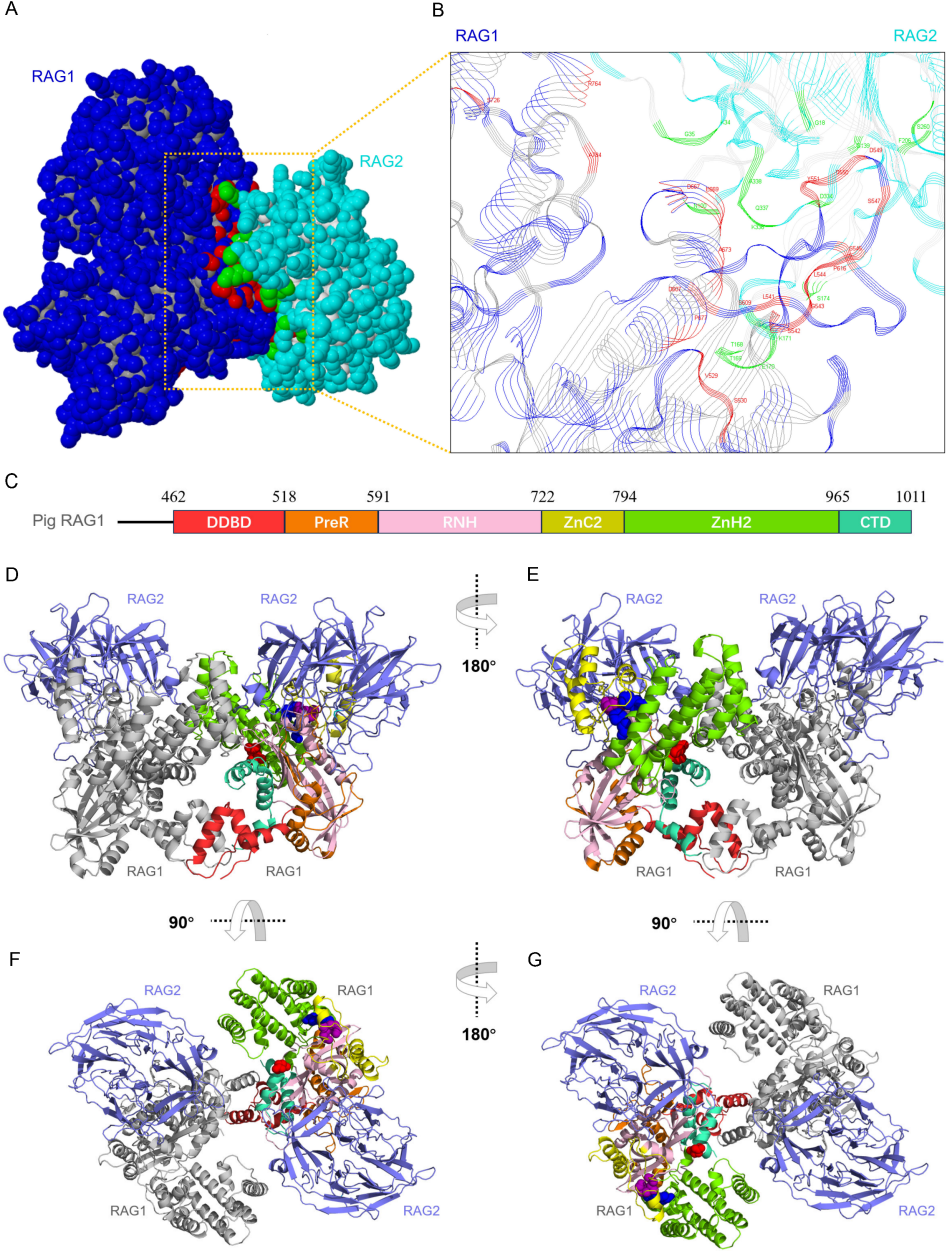
Predicted pig RAG1-RAG2 heterodimer and heterotetramer structures. **(A)** Cartoon representation of the predicted pig RAG1-RAG2 heterodimer obtained by molecular docking. **(B)** Detailed view of the heterodimerization interface. The interface region (shown as strands) is separated and rotated for clarity. Pig RAG1 is colored blue/gray, and pig RAG2 is colored cyan/light gray. Interface residues are highlighted in red (RAG1) and chartreuse (RAG2). **(C)** Schematic representation of RAG1 domain organization with boundaries indicated by residue numbers. **(D)** Cartoon representation of the predicted pig RAG1-RAG2 heterotetramer. One RAG1 subunit is colored as in **(C)**, the second RAG1 subunit is shown in silver, and RAG2 is shown in slate. E965 is highlighted using red spheres. The zinc-binding residues C730/C733 are shown as hot-pink spheres, and H940/H945 as blue spheres. **(E)** Rotated view (180°) of the pig RAG1-RAG2 heterotetramer. **(F, G)** Additional rotated views (90°) of panels **(D, E)**, respectively.

Because RAG1 and RAG2 function cooperatively in DNA binding and cleavage, we performed a prediction and analysis of pig RAG1-RAG2 heterodimerization. Protein–protein docking analysis indicated that pig RAG1 and RAG2 were able to form heterodimers (**Figure 4A**). PDBePIA revealed that the heterodimeric interfaces of pig RAG1–RAG2 were predominantly formed by residues within highly conserved core regions (**Figure 4B**; **Supplementary Tables 2**, **3**).

Additionally, we further modeled the RAG1-RAG2 heterotetramer. The predicted structure has a Y-shaped configurate. The RAG1 dimer forms the main body, whereas RAG2 sits at the tip of each arm (**Figures 4D–G**). Each active site is located midway along the arm of the Y-shaped structure. Between D711 and E965 lie two structurally critical domains: ZnC2 (highlighted in yellow), which protrudes toward RAG2, and the helical ZnH2 domain (highlighted in chartreuse), which contributes to the 3D architecture of RAG1 and positions E965 (shown as red sticks) within the catalytic center (**Figures 4C–G**). The two zinc-binding regions—C730/C733 (hot-pink sticks) and H940/H945 (blue sticks)—form a unified zinc site adjacent to the catalytic core. This site bridges functionally distinct regions, namely, the RAG2 interface (ZnC2), the DNA-binding site (DDBD/ZnH2), and the catalytic core, despite the linear separation of these residues in the primary sequence. Multiple RAG2 loops, particularly those linking adjacent β-propeller blades and the central strands within each blade, engage extensively with the preR, RNH, and ZnC2 domains of RAG1.

### 3.5 Changes in the catalytic core domains of RAG1 and RAG2 cause immunodeficiency in pig models

Evidence from previous studies, together with our own experimental models, indicates that changes in the catalytic core domains of *RAG1* and *RAG2* cause immunodeficiency in pigs.

**Figure 5:**
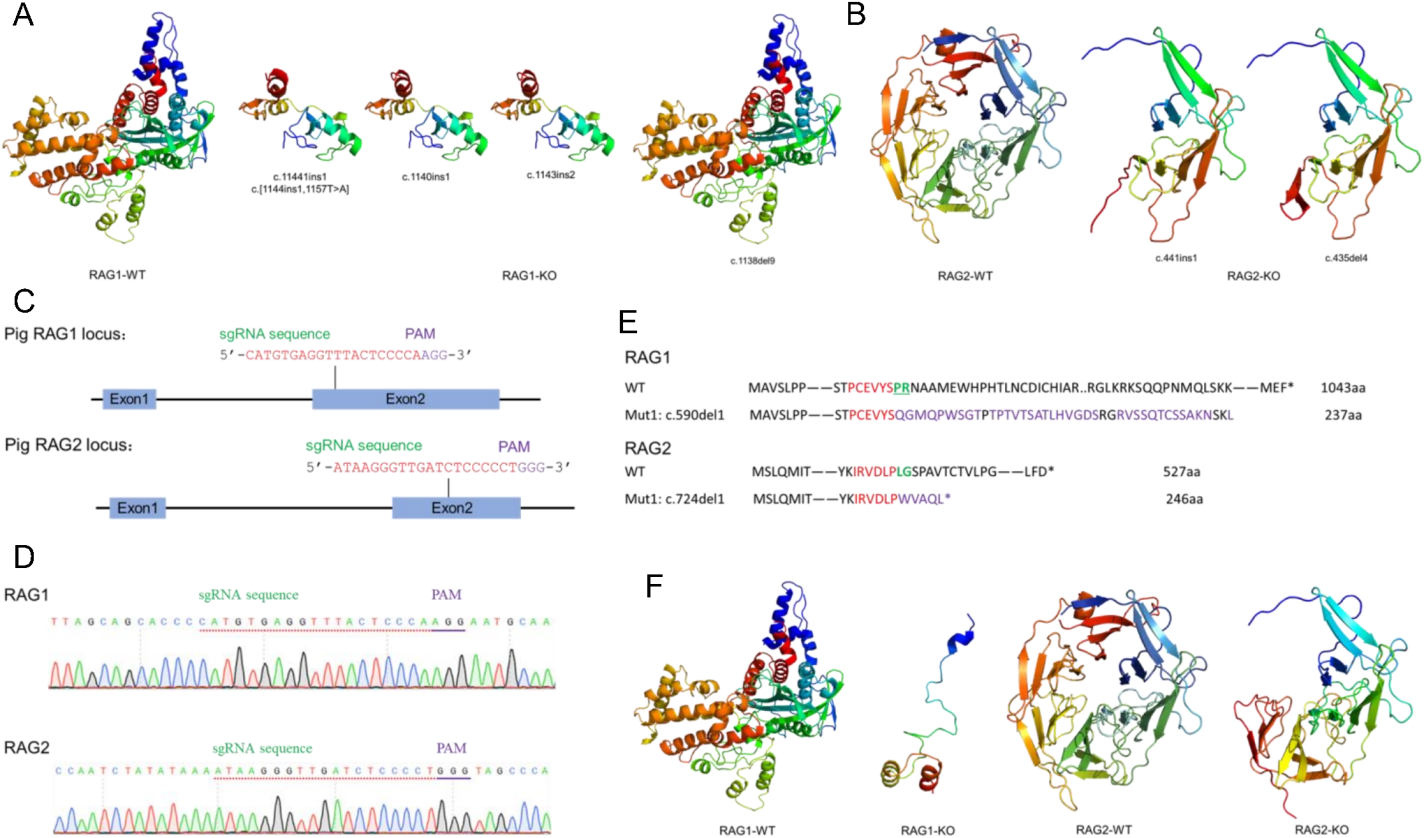
Predicted structural consequences of *RAG1* and *RAG2* in knockout immunodeficient pig models. **(A)** Predicted structural effects of RAG1 mutations in *RAG1/TYR* KO Tibet minipigs. Mutant RAG1 lacks multiple α-helices and all β-strands within the affected region. **(B)** Predicted structural consequences of RAG2 mutations in *RAG2* KO miniature pigs. Mutant RAG2 lacks all α-helices and part of the β-strands architecture. **(C)** Target sites for *RAG1* and *RAG2* gene editing in *RAG1/RAG2* DKO pig. Genomic sequences showing the sgRNA protospacer regions (highlighted in red) and adjacent PAM sequences (highlighted in purple) for both loci. **(D)** Validation of RAG1 and RAG2 gene editing in *RAG1/RAG2* DKO pig. Sanger sequencing chromatograms of modified RAG1 and RAG2 genomic regions encompassing the sgRNA target sites. Protospacer sequences are indicated by red dashed underlines; adjacent PAM sequences are marked by purple solid underlines. **(E)** Impact of editing on RAG1 and RAG2 protein sequences in *RAG1/RAG2* DKO pig. The schematic representation shows amino acid (AA) sequence alterations at target sites in KO piglets. The AA residues encoded by the protospacer region are shown in red text. Residues encoded by the PAM sequence region are highlighted in green and underlined. The modified AA residues resulting from the edits are highlighted in purple. **(F)** Predicted structural consequences of RAG1 and RAG2 mutations in *RAG1/RAG2* DKO pig. Mutant RAG1 lacks portions of α-helices and all β-strands, whereas mutant RAG2 lacks all α-helices and portions of β-strands structures.

Chen et al. (56) generated *RAG1/TYR* knockout (KO) Tibet minipigs presenting typical phenotypes of immunodeficiency and albinism, which was characterized by abnormal immunocyte proportions and thymic atrophy. The four *RAG1* knockout (KO) pigs were found to carry five different *RAG1* mutations: c.11441ins1, c.[1144ins1,1157T>A], c.1140ins1, c.1143ins2, and c.1138del9. These mutations gave rise to four distinct truncated proteins. Among them, the c.1138del9 mutation led to a loss of only three amino acids in a non-core region. Conversely, the remaining three mutations resulted in the deletion of the catalytic core domains of RAG1. 3D structural analysis indicated that the absence of key domains in RAG1 (including pre-R, RNH, and ZnC2) prevented extensive binding with RAG2, thereby compromising the function of the RAG complex (**Figure 5A**). Further characterization of the four mutant Tibetan miniature pigs revealed that the individual with only a three-amino-acid deletion in the non-core region exhibited no immunological deficiencies, whereas the others all displayed severe phenotypes of abnormal immunocyte proportions and thymic atrophy. These findings are consistent with our initial hypothesis that changes in the catalytic core domains of RAG1 and RAG2 cause immunodeficiency in pig models.

Similarly, Wang et al. (35) created *RAG2* KO immune-deficient miniature pigs that were characterized by significantly reduced thymus and spleen size, as well as a markedly lower proportion of mature T and B lymphocytes, relative to wild-type controls. The *RAG2* KO pigs carried two distinct mutations (c.441ins1 and c.435del4), both of which resulted in the production of truncated proteins of 173 amino acids in length. These truncated proteins lost the majority of the β-propeller domain and the entire PHD domain. Structural analysis revealed that the mutant RAG2 contained only two complete kelch repeats (**Figure 5B**). The absence of critical loops prevented RAG2 from forming extensive interactions with the pre-R, RNH, and ZnC2 domains of RAG1, thereby compromising its function.

**Figure 6:**
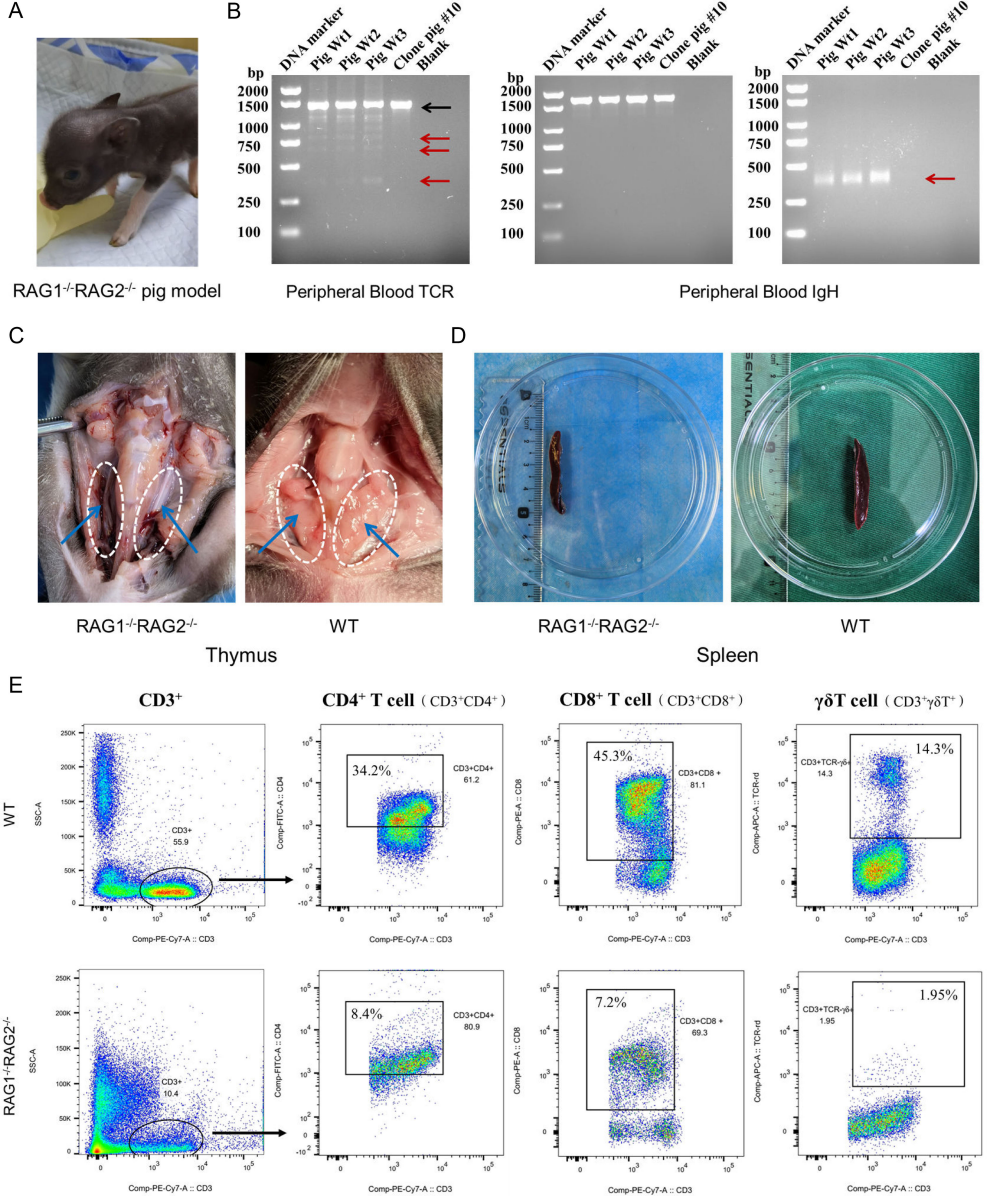
Catalytic core mutations in pig RAG1 and RAG2 lead to immunodeficiency in the pig model. **(A)** Representative image of the *RAG1/RAG2* DKO pig model. **(B)** Analysis of V(D)J recombination at the TCRβ and BCR loci in the PB of the *RAG1/RAG2* DKO pig model. PCR results of the TCRβ locus revealed only germline bands (black arrow) on the gel in DKO pigs, in contrast with WT pigs, which presented additional, shortened rearranged bands (red arrows). The rearranged bands (red arrow) at the IgH locus were also detected only in the WT pigs. **(C)** Anatomical structure of the thymus in the *RAG1/RAG2* DKO pig model. DKO pigs completely lacked thymic tissue, which is highlighted by white dotted circles and blue arrows, whereas age-matched WT controls displayed normal thymuses. **(D)** Anatomical structure of the spleen in the *RAG1/RAG2* DKO pig model. The spleens of DKO pigs were smaller than those of age-matched WT pigs. **(E)** The proportions of CD4^+^ T cells, CD8^+^ T cells and γδ T cells in the peripheral blood of *RAG1*/*RAG2* DKO piglet were determined by flow cytometry.

In addition, we generated *RAG1/RAG2* DKO pigs using CRISPR/Cas9-mediated editing and SCNT. Positive individuals were identified using PCR and Sanger sequencing of blood-derived genomic DNA (**Figure 5D**). The mutations in amino acid sequences and the predicted consequences for 3D protein structures are shown in **Figures 5E, F**. The mutations resulted in the deletion of the catalytic core domains of RAG1 and more than two complete kelch repeats of RAG2, which resulted in the deletion of RAG1-RAG2 heterodimers and heterotetramers, and thereby affecting the function. The *RAG1/RAG2* DKO pig exhibited immunodeficiency (**Figure 6**), which was characterized by the fact that no V(D)J rearrangement was detected in the peripheral blood (PB) of *RAG1/RAG2* DKO piglet at the TCRβ and BCR IgH loci (**Figure 6B**). Compared to the control piglets, *RAG1/RAG2* DKO piglet showed thymus deletion and significantly smaller spleen (**Figures 6C, D**). We also performed flow cytometric analysis of the proportion of T cells in peripheral blood, and the results demonstrated markedly reduced proportions of CD4^+^ T cells, CD8^+^ T cells and γδ T cells in *RAG1*/*RAG2* DKO piglet (**Figure 6E**).

## 4 Discussion

RAG1 and RAG2 proteins are essential mediators of V(D)J recombination during lymphocyte development, and their evolutionary origin and functional interplay have been extensively investigated. In this study, we integrated evolutionary, structural, and functional analysis to characterize RAG1 and RAG2 in vertebrates, with particular emphasis on pigs. These analyses informed the rational development of *RAG1/RAG2*-deficient pig models for immunological research.

By performing a direct evolutionary and structural-functional correlation analysis, we demonstrate that mutations affecting specific domains, particularly the catalytic core of RAG1 and the PHD domains of RAG2, abolish V(D)J recombination in pigs. Evolutionary and structural analyses revealed high conservation of the RAG1 core region and RAG2 β-propeller/PHD domains, indicating that these regions evolve slowly and that evolutionary conservation has dictated functional constraints in the RAG complex architecture. Our phylogenetic analysis of 132 vertebrate RAG1/RAG2 homologs provides further supports the obligate partnership of RAG1 and RAG2. First, their highly congruent phylogenies indicate tightly coupled co-evolution, consistent with their origin as a *Transib*-derived gene cluster. While RAG1 shares ancestry with *Transib* transposases, RAG2 has evolved novel epigenetic functions of the PHD through synergistic co-option, which is a process that is conserved from teleosts to mammals, underscoring its obligate partnership in adaptive immunity. Additional studies have indicated that RAG2 destroys the condensed state of RAG1 through direct interaction with RAG1 to activate robust V(D)J recombination without relying on the PHD functional domain of RAG2 (57). The functions of the noncore region of RAG2 require further investigation. Second, ConSurf evolutionary rate mappings of RAG1 and RAG2 identified molecular “fossils”, which are ultra-conserved residues under strong purifying selection. For example, RAG1’s catalytic core (aa 387–1011) includes the catalytic triad (D603/D711/E965) and zinc-coordinating residues (C730/C733; H940/H945), as does RAG2’s β-propeller scaffold (aa 1–352) and epigenetic PHD finger (aa 414–491). These regions represent evolutionary “hard constraints” essential for V(D)J recombination, and the high degree of conservation observed in these regions across vertebrates highlights their importance in the biological functions of the proteins. Finally, the comparative immunology study by Sinkora et al. (58) confirmed that pig Ig gene rearrangement follows the same core rules as mice and humans, and these processes all rely on the recognition and catalysis of RSS by RAG1/2. This also directly proves that the core catalytic function and RSS recognition mechanism of pig RAG1/2 are related to other mammals and are highly conserved, creating a functional basis for their structural analysis. The core domains of pig RAG1/2 (such as the catalytic core region of RAG1 and the PHD of RAG2) are highly homologous to mice and humans and are the molecular guarantee for maintaining the core rules of Ig rearrangement. In addition, based on the macroevolutionary background of the rules governing the immunoglobulin rearrangement mechanism revealed by comparative immunology (58), the core catalytic domain of pig RAG1/2 retains the ancestral characteristics of vertebrates (highly homologous to those of amphibians, fish, and mice), while variations in its regulatory regions and substrate recognition domains are the results of adaptive evolution to the rearrangement pattern where IgL precedes IgH in pigs.

A comprehensive structural analysis of pig RAG1 and RAG2 further supported the functional significance of their conserved domains, which not only complements the evolutionary understanding of RAG proteins across species but also lays a critical foundation for future studies on the molecular mechanism of RAG-mediated V(D)J recombination in pigs. 3D modeling of pig RAG1 and RAG2 revealed that the catalytic triad and other conserved residues cluster, facilitating the interaction between RAG1 and RAG2. The predicted heterodimeric and heterotetrameric structures of pig RAG1-RAG2 suggest that these proteins can form stable complexes, which are essential for their DNA binding and cleavage activities. The interfaces of these complexes are predominantly formed by residues within highly conserved core regions, highlighting the importance of these regions in protein–protein interactions. Heterodimer/heterotetramer docking analyses demonstrated that pig RAG1-RAG2 complexes adopt configurations analogous to those of human orthologs, supporting their capacity to execute DNA cleavage and recombination. This structural congruence provides rationale for establishing pigs as pathophysiologically relevant models for RAG deficiency. However, the tertiary structure and RAG1-RAG2 protein docking of pig in this study were predicted on the basis of the core structure of other species, and the expression of the noncore regions of the pig RAG1 and RAG2 proteins needs further investigation. Also, as the rearrangement of pig IgL occurs before that of IgH, suggesting that there is species specificity in the expression timing of pig *RAG1*/*2* during the early stage of B cell development — it is activated at the IgL locus earlier than at the IgH locus (58). This finding offers a new outlook for analyzing the structural characteristics of the pig RAG1/2 promoter and regulatory regions, as well as the differences in their binding to transcription factors, from the perspectives of transcriptional regulation and epigenetic modifications (such as histone modifications).

Defective DNA processing in V(D)J recombination drives SCID or a milder type of immunodeficiency, Omenn syndrome. Over 60 causative missense mutations in the catalytic cores of *RAG1/RAG2* have been reported to cause SCID or Omenn syndrome (59). The mutations causing SCID and Omenn syndrome are divided into four categories. The first type of mutation occurs in two DNA-interaction regions of DDBD/CTD and NBD domains and is characterized by the solvent-exposed polar residues. For example, mutations of positively charged residues (e.g., K969A, R972A, R980A, H993A, residues numbered based on the pig protein) in RAG1-CTD disrupt DNA binding and cleavage (**Supplementary Figure 1**) (60). Some mutations localize to evolutionarily conserved polar/nonpolar residues, compromising both structural stability and sequence-specific nonamer DNA recognition (5, 61). The second type of mutation clusters near the active site. Certain mutations potentially disrupt the structure of the catalytic center (S601P, C602W, A622P, R699Q/W, and G709D), whereas others might modify its DNA-binding characteristics (E669G and R624C/H) (60, 62). The third category of mutations disturbs the tertiary structure of RAG1-RAG2. For example, mutations of C730E and L733F at the zinc-binding site disrupt ZnC2, ZnH2 and adjacent domains, and mutations of W896R, Y912C and I956R disrupt the hydrophobic core proximal to this zinc-binding motif, underscoring its critical role in RAG1-RAG2 complex stability (5). The final category of mutations occurs at the RAG1-RAG2 interface (5, 63). The importance of the interface is highlighted by disease mutations on RAG1 (R559S, R561C/H, E669G, R776Q) and additional laboratory-identified mutations (E722Q, R776A) (60, 62). All of these cases of disease caused by various mutations in the core region highlight the critical importance of this region’s function. By comparing the RAG mutations responsible for human SCID/Omenn syndrome with the pig RAG ortholog and performing cross-species structural conservation analysis, we identified that these sites are highly conserved.

The functional validation of RAG1 and RAG2 in pigs was demonstrated through the creation of *RAG1/RAG2* DKO pigs using CRISPR/Cas9-mediated editing and SCNT. The immunodeficiency observed in these animals, characterized by the absence of V(D)J rearrangements in the TCRβ and IgH loci, thymic absence and smaller spleens, as well as a significantly decrease in the proportions of CD4^+^ T cells, CD8^+^ T cells and γδ T cells, strongly supports the critical role of RAG1 and RAG2 in adaptive immunity. These results are consistent with the known functions of RAG1 and RAG2 in V(D)J recombination and further validate the importance of these proteins in the development of the immune system. However, due to severe damage to the immune system, SCID animals have strict requirements for feeding conditions, which greatly limits the life span of immunodeficient animals. Our *RAG1*/*RAG2* DKO piglet was raised in a conventional housing environment, and developed fever and pulmonary edema 3 days after birth, consistent with previously reported phenotypes (**Table 1**) (11, 12, 64–66). Therefore, as an animal model of human disease, *RAG1/RAG2* DKO pigs need to be improved in the long-term exploration of human diseases. Our findings provide a critical basis for subsequent xenotransplantation studies (human tumor cell/tissue transplantation) for establishing of humanized pig models for advancing immunological research and therapy (30). Similarly, Ren et al. (67) generated *FAH*-deficient and severe combined immunodeficient pig models (*FAH*/*RAG1* DKO and *FAH*/*RAG1*/*IL2RG* triple knockout) to evaluate chronic liver injury and severe immunodeficiency. Primary human hepatocytes were successfully transplanted into *FAH*/*RAG1* (FG) pigs, yielding stable liver-humanized pigs with time-dependent human albumin expression as early as 1-week post-transplantation. This model provides a valuable platform for studying hepatocyte regeneration and hepatocyte-based therapies, creating a basis for humanized organ reconstruction. Pi et al. (31) introduced the newly generated immunodeficient RGD pigs deficient in *RAG1*, *IL2RG*, and *CD47*, which support efficient, long-term multilineage human hematopoietic reconstitution following transplantation of human hematopoietic stem/progenitor cells (HSPCs), representing a promising preclinical model for studying human hematopoiesis, related therapies, and serving as a potential bioreactor for the large-scale generation of human immune cells. In addition, Waide et al. (68) first characterized a naturally occurring SCID Yorkshire pig model, identifying two independent mutations in the *Artemis* gene(g.51578763 G→A resulting in a truncated protein lacking 47 amino acids and g.51584489 G→A leading to a truncated protein). This model offers a valuable resource for investigating the pathogenesis, genotype–phenotype correlations, and potential “leaky” phenotypes associated with human Artemis-SCID, thereby enhancing our understanding of how distinct genetic lesions can lead to immune deficiency. The group further generated *ART*^–/–^*IL2RG*^–/Y^ pigs with a T^–^B^–^NK^–^ phenotype using CRISPR/Cas9 (69), providing critical insights and inspiration for our future research endeavors.

**Table 1:** Survival data and morbidity details of RAG1/2 KO pigs.

Genotype	Immune phenotype	Rearing method	Survivals	Morbidity details
RAG1^-/-^	T ^–^ B ^–^	Did not rear	Neonatal	Deficient immune function (70)
RAG2^+/-^	T ^–^ B ^–^	Conventional housing	Survived until reached sexual maturity	No obvious infection or inflammation (Study observation endpoint) (12)
RAG1^-/-^	T ^–^ B ^–^	Conventional housing	<29 days	Slower weight gain and deficient immune function (12)
	T ^–^ B ^–^	Clean/Barrier environment	>8 weeks	No obvious infection or inflammation (Study observation endpoint) (12)
RAG2^-/-^	T ^–^ B ^–^ NK ^+^	Conventional housing	<29 days	Failure to thrive; elevated body temperature accompanied by trembling; spleen inflammation and cell apoptosis, and died of systemic inflammation (64)
	T ^–^ B ^–^	Conventional housing	<21 days	Slower weight gain and deficient immune function (12)
	T ^–^ B ^–^	Conventional housing	<35 days	Reduced immunity caused some inflammatory reactions (35)
	T ^–^ B ^–^ NK ^+^	Conventional housing	12 weeks	Euthanized (71)
	T ^–^ B ^–^	Clean/Barrier environment	>8 weeks	No obvious infection or inflammation (Study observation endpoint) (12)
RAG1^-/-^IL2RG^-/-^	T ^–^ B ^–^ NK ^–^	SPF environment	>500 days	Not mentioned (72)
RAG1^-/-^TYR^+/-^ or RAG1^-/-^TYR^-/-^	T ^–^ B ^–^ NK ^+^	Clean environment	95 days-4.5 months	Colon cancer and metastatic lesions in the spleen; multiple infections or unexplained weight loss (not excluding potential infection) (56)
RAG1^-/-^ RAG2^-/-^IL2RG^-/-^	T ^–^ B ^–^ NK ^–^	Conventional housing	49 days	Infection in the lung (11)
RAG1^+/-^ RAG2^-/-^IL2RG^-/-^	T ^–^ B ^–^ NK ^–^	Conventional housing	12 days	Infection in the lung (11)
RAG2^-/-^IL2RG^-/-^	T ^–^ B ^–^ NK ^–^	Gnotobiotic isolator	34 days	Depletion of lymphocytes and either absence of or structurally abnormal immune organs (66)
RAG2^+/-^IL2RG^+/-^ or RAG2^-/-^IL2RG^+/-^	T ^–^ B ^–^ NK ^–^	Gnotobiotic isolator	<8 weeks	Discitis; fever and arthritis; persistent infection (65)
RAG2^-/-^IL2RG^y^/^-^	T ^–^ B ^–^ NK ^–^	Conventional housing	21 days	Study observation endpoint (73)
FAH^−/−^RAG1^−/−^IL2RG^−/Y^	T ^–^ B ^–^ NK ^–^	Isolator	>5 months	Data not shown (67)
RAG2^-/-^IL2RG^+/-^	T ^–^ B ^–^ NK ^–^	Conventional housing	41 and 75 days	Infection in the lung (11)

## Data Availability

The original contributions presented in the study are included in the article/**Supplementary Material**. Further inquiries can be directed to the corresponding authors.

## Supplementary material

Supplementary Table 1 Sequence data used in this study (provided as an Excel file).

Supplementary Table 2 Interfacing residues of RAG1 in pig RAG1-RAG2.

Supplementary Table 3 Interfacing residues of RAG2 in pig RAG1-RAG2.

Supplementary Table 4 Primers used in this study.

Supplementary Figure 1 Characteristics of hydrophilicity and polarity in the secondary structure of pig RAG1 predicted by PSIPRED.
