# Supplementary material for: Evolutionary insights and structural characterization guide the development of RAG1/RAG2-deficient swine models for immunological research
Source: Front Immunol. 2026 Mar 25;17:1757508. doi: 10.3389/fimmu.2026.1757508 (PMC13056857; doi:10.3389/fimmu.2026.1757508)
Supplement: Supplementary Table 1 — Sequence data used in this study (provided as an Excel file). [file DataSheet1.docx]

Supplementary Material

# Supplementary Figures


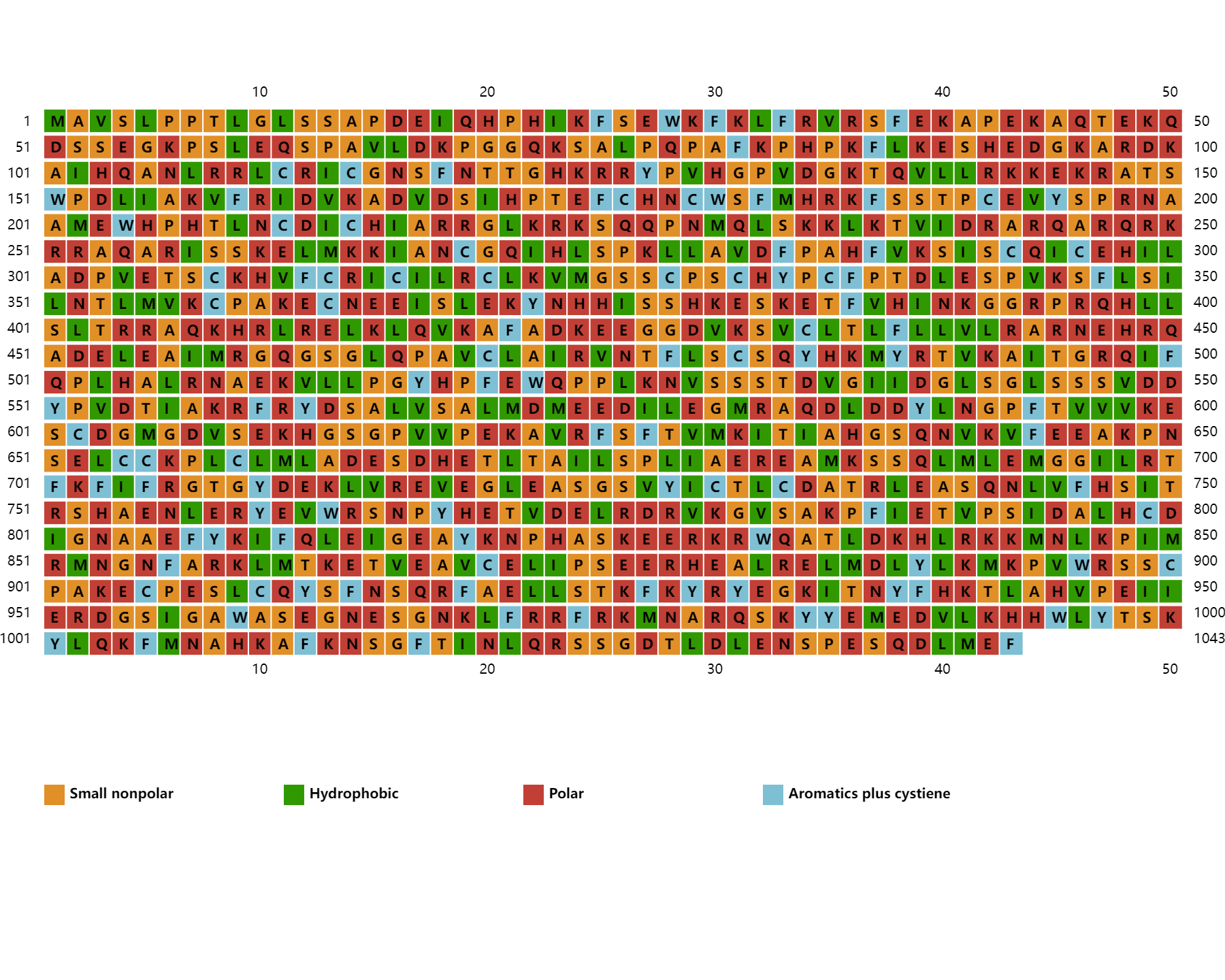


**Supplementary Figure 1.** Characteristics of hydrophilicity and polarity in the secondary structure of pig RAG1 predicted by PSIPRED.

# Supplementary Tables

**Supplementary Table S1.** Sequence data used in this study.

| **Class** | **Order** | **Suborder** | **Species** | | | ***RAG1*** | ***RAG2*** |
| --- | --- | --- | --- | --- | --- | --- | --- |
|  |  |  | **Family** | **Scientific name** | **Common name** |  |  |
| **Pisces** | **Cypriniformes** |  | **Characin** | ***Astyanax mexicanus*** | **Mexican tetra** | XP_022527882 | XP_007228210 |
|  |  |  | **Cyprinidae** | ***Danio rerio*** | **zebrafish** | NP_571464.1 | NP_571460.2 |
|  | **Siluriformes** |  | **Ictaluridae** | ***Ictalurus punctatus*** | **channel catfish** | XP_017340081.1 | XP_053541630.1 |
|  | **Salmoniformes** |  | **Salmonidae** | ***Oncorhynchus mykiss*** | **rainbow trout** | NP_001118209.1 | XP_021428825.1 |
|  |  |  |  | ***Oncorhynchus kisutch*** | **coho salmon** | XP_020312197.1 | XP_020312042.1 |
|  |  |  |  | ***Salmo salar*** | **Atlantic salmon** | XP_013980582.1 | N/A |
|  | **Beloniformes** |  | **Adrianichthyidae** | ***Oryzias latipes*** | **Japanese medaka** | XP_023811703.1 | XP_004069774 |
|  | **Perciformes** | **Labroidei** | **Pomacentridae** | ***Amphiprion ocellaris*** | **Amphiprion nigripes** | N/A | N/A |
|  |  |  | **Gobiidae** | ***Boleophthalmus pectinirostris*** | **great blue-spotted mudskipper** | N/A | XP_020786787.1 |
|  |  |  | **Labridae** | ***Labrus bergylta*** | **ballan wrasse** | XP_065813388.1 | XP_020498570.1 |
|  | **Synbgranchiformes** |  | **Synbranchidae** | ***Monopterus albus*** | **swamp eel** | XP_020470881.1 | XP_020470878.1 |
|  | **Chimaeriformes** |  | **Callorhinchidae** | ***Callorhinchus milii*** | **elephant fish** | XP_007886047.1 | XP_007885835.1 |
| **mammals** | **Cetacea** | **Mysticeti** | **Balaenopteridae** | ***Balaenoptera acutorostrata*** | **Minke whale** | XP_007181154.2 | XP_007181156.1 |
|  |  | **Odontoceti** | **Physeteridae** | ***Physeter catodon*** | **Sperm whale** | XP_007106929.2 | XP_007106924.1 |
|  |  |  | **Lipotidae** | ***Lipotes vexillifer*** | **Baiji** | XP_007466909.1 | XP_007466911.1 |
|  |  |  | **Delphinidae** | ***Orcinus orca*** | **Killer whale** | XP_004264065.1 | XP_004264066.1 |
|  |  |  |  | ***Tursiops truncatus*** | **Bottlenose dolphin** | XP_004329867.3 | XP_019803923.1 |
|  |  |  | **Monodontidae** | ***Monodon monoceros*** | **Narwhal** | XP_029065553.1 | XP_029065143.1 |
|  |  |  |  | ***Delphinapterus leucas*** | **beluga whale** | XP_022439312.1 | XP_022439308.1 |
| **Reptilia** | **Squamata** | **Sauria** | **Family   Agamidae** | ***Pogona vitticeps*** | **central bearded dragon** | XP_020669497.1 | XP_020669498.1 |
| **mammals** | **Artiodactyla** |  | **Bovidae** | ***Ovis aries*** | **Sheep** | XP_004016460.1 | XP_060256044.1 |
|  |  |  |  | ***Capra hircus*** | **Goat** | XP_005690149.2 | XP_005690152.1 |
|  |  |  |  | ***Bubalus bubalis*** | **water buffalo** | XP_006062402.2 | XP_006062397.1 |
|  |  |  |  | ***Bos taurus*** | **Cow** | XP_024831670.1 | NP_001075938.1 |
|  |  |  | **Cervidae** | ***Odocoileus virginianus texanus*** | **White-tailed Deer** | XP_020760624.1 | XP_020760626.1 |
|  |  | **Tylopoda** | **Camelidae** | ***Camelus dromedarius*** | **Dromedary Camel** | XP_010998899.1 | XP_031315641.1 |
|  |  |  | **Suidae** | ***Sus scrofa*** | **Pig** | NP_001116656.1 | NP_001121953.1 |
| **Aves** | **Passeriformes** | **Passerii** | **Fringillidae** | ***Serinus canaria*** | **Canary** | XP_009100398.1 | XP_030096686.2 |
|  |  |  | **Corvidae** | ***Corvus cornix cornix*** | **Hooded Crow** | XP_039408870.1 | XP_010405548.2 |
|  |  |  | **Estrildidae** | ***Lonchura striata domestica*** | **Bengalese finch** | XP_021410775.1 | XP_021410793.1 |
|  | **Galliformes** |  | **Phasianidae** | ***Meleagris gallopavo*** | **Turkey** | XP_010709561.1 | N/A |
|  |  |  |  | ***Gallus gallus*** | **chicken** | NP_001026359.2 | NP_001291986.1 |
|  |  |  | **Numididae** | ***Numida meleagris*** | **helmeted guineafowl** | XP_021259573.1 | XP_021257747.1 |
|  | **Anseriformes** |  | **Anatidae** | ***Anas platyrhynchos*** | **mallard** | N/A | XP_038036182.1 |
|  | **Struthioniformes** |  | **Dromaiidae** | ***Dromaius novaehollandiae*** | **Emu** | XP_025953487.2 | XP_025953472.1 |
|  | **Columbiformes** |  | **Columbidae** | ***Columba livia*** | **rock pigeon** | XP_005500299.3 | XP_064920909.1 |
| **mammals** | **Perissodactyla** |  | **Equidae** | ***Equus caballus*** | **Horse** | NP_001243830.1 | XP_014584913.1 |
|  | **Carnivora** |  | **Mustelidae** | ***Mustela putorius furo*** | **Ferret** | XP_004756059.2 | XP_004756065.1 |
|  |  |  | **Phocidae** | ***Leptonychotes weddellii*** | **Weddell Seal** | XP_006736643.1 | XP_006736644.1 |
|  |  |  | **Canidae** | ***Vulpes vulpes*** | **Red Fox** | XP_025868719.1 | XP_025868535.1 |
|  |  |  |  | ***Canis familiaris*** | **Dog** | XP_038279430.1 | XP_038280005.1 |
|  |  |  | **Ursidae** | ***Ursus arctos horribilis*** | **Grizzly** | N/A | N/A |
|  |  |  | **Phocidae** | ***Neomonachus schauinslandi*** | **Hawaiian monk seal** | XP_021540423.2 | XP_021540424.1 |
|  |  |  | **Felidae** | ***Felis catus*** | **Cat** | XP_019667845.1 | XP_004001473.3 |
|  | **Chiroptera** |  | **Pteropodidae** | ***Pteropus vampyrus*** | **Megabat** | N/A | N/A |
|  |  |  | **Vespertilionidae** | ***Myotis lucifugus*** | **Microbat** | N/A | N/A |
|  | **Primates** |  | **Hominidae** | ***Homo sapiens*** | **Human** | NP_001364209.1 | NP_001230715.1 |
|  |  |  |  | ***Pan troglodytes*** | **Chimpanzee** | XP_016776175.1 | XP_054518186.2 |
|  |  |  |  | ***Gorilla gorilla gorilla*** | **Gorilla** | XP_055211515.2 | XP_004051008.3 |
|  |  |  |  | ***Pongo abelii*** | **Orangutan** | XP_063527055.1 | XP_054296934.2 |
|  |  |  | **Hylobatidae** | ***Nomascus leucogenys*** | **Gibbon** | N/A | N/A |
|  |  |  | **Cercopithecidae** | ***Macaca nemestrina*** | **Pig-tailed macaque** | XP_011722423.1 | XP_011722426.1 |
|  |  |  |  | ***Papio anubis*** | **Olive baboon** | XP_003910066.2 | XP_003910067.1 |
|  |  |  |  | ***Macaca mulatta*** | **Macaque** | NP_001253701.1 | XP_001114748.3 |
|  |  |  | **Lemuridae** | ***Microcebus murinus*** | **gray mouse lemur** | XP_012594188.1 | XP_012594181.1 |
|  |  |  | **Tarsiidae** | ***Carlito syrichta*** | **Philippine tarsier** | XP_008059534.1 | XP_008059536.1 |
|  |  |  | **Callitrichidae** | ***Callithrix jacchus*** | **Marmoset** | XP_009006192.1 | XP_035118274.1 |
|  |  |  | **Galagidae** | ***Otolemur garnettii*** | **small-eared galago** | XP_023365072.1 | XP_023365081.1 |
|  | **Rodentia** |  | **Muridae** | ***Mus musculus*** | **Mouse** | NP_033045.2 | NP_033046.1 |
|  |  |  |  | ***Rattus norvegicus*** | **Rat** | NP_445920.1 | NP_001093998.1 |
|  |  |  |  | ***Mus pahari*** | **shrew mouse** | XP_021050462.1 | XP_021050297.1 |
|  |  |  |  | ***Mus caroli*** | **Ryukyu mouse** | XP_029330443.1 | XP_021041956.1 |
|  |  |  | **Sciuridae** | ***Ictidomys tridecemlineatus*** | **Squirrel** | XP_005327451.1 | XP_005327449.1 |
|  |  |  | **Cricetidae** | ***Mesocricetus auratus*** | **golden hamster** | XP_012976182.1 | XP_021087232.1 |
|  |  |  |  | ***Meriones unguiculatus*** | **Mongolian gerbil** | XP_021507572.1 | XP_021507574.1 |
|  |  |  | **Bathyergidae** | ***Heterocephalus glaber*** | **naked mole-rat** | XP_004852072.1 | XP_004852071.1 |
|  |  |  | **Castoridae** | ***Castor canadensis*** | **American beaver** | XP_020030509.1 | XP_020030508.1 |
|  |  |  | **Caviidae** | ***Cavia porcellus*** | **Guinea pig** | XP_003463881.1 | XP_023418210.2 |
|  | **Didelphimorphia** |  | **Didelphidae** | ***Monodelphis domestica*** | **gray short-tailed opossum** | XP_056659760.1 | XP_001362290.2 |
|  | **Diprotodontia** |  | **Phascolarctidae** | ***Phascolarctos cinereus*** | **koala** | XP_020819598.1 | XP_020819600.1 |
|  | **Lagomorpha** |  | **Leporidae** | ***Oryctolagus cuniculus*** | **Rabbit** | NP_001164611.1 | NP_001164612.1 |
|  | **Cingulata** |  | **Dasypodidae** | ***Dasypus novemcinctus*** | **Armadillo** | XP_058161537.1 | XP_004483231.2 |
|  | **Proboscidea** |  | **Elephantidae** | ***Loxodonta africana*** | **African elephant** | XP_003412322.1 | XP_023406471.1 |

**Supplementary Table S2.** Interfacing residues of RAG1 in RAG1-RAG2.

| **Num** | [**Structure 1**](javascript:openWindow('pi_ipage_res1.html',400,250);) | [**HSDC**](javascript:openWindow('pi_ipage_hs.html',400,250);) | [**ASA**](javascript:openWindow('pi_ipage_asa.html',400,250);) | [**BSA**](javascript:openWindow('pi_ipage_bsa.html',400,250);) | [**Δ**^i^**G**](javascript:openWindow('pi_ipage_rdg.html',400,250);) |
| --- | --- | --- | --- | --- | --- |
| 1 | A:SER 463 |  | 108.15 | 0.00 | 0.00 |
| 2 | A:GLY 464 |  | 46.83 | 0.00 | 0.00 |
| 3 | A:LEU 465 |  | 55.51 | 0.00 | 0.00 |
| 4 | A:GLN 466 |  | 102.59 | 0.00 | 0.00 |
| 5 | A:PRO 467 |  | 49.10 | 0.00 | 0.00 |
| 6 | A:ALA 468 |  | 18.17 | 0.00 | 0.00 |
| 7 | A:VAL 469 |  | 29.02 | 0.00 | 0.00 |
| 8 | A:CYS 470 |  | 0.00 | 0.00 | 0.00 |
| 9 | A:LEU 471 |  | 1.51 | 0.00 | 0.00 |
| 10 | A:ALA 472 |  | 0.33 | 0.00 | 0.00 |
| 11 | A:ILE 473 |  | 48.02 | 0.00 | 0.00 |
| 12 | A:ARG 474 |  | 14.90 | 0.00 | 0.00 |
| 13 | A:VAL 475 |  | 12.43 | 0.00 | 0.00 |
| 14 | A:ASN 476 |  | 25.52 | 0.00 | 0.00 |
| 15 | A:THR 477 |  | 60.84 | 0.00 | 0.00 |
| 16 | A:PHE 478 |  | 70.30 | 0.00 | 0.00 |
| 17 | A:LEU 479 |  | 58.49 | 0.00 | 0.00 |
| 18 | A:SER 480 |  | 15.92 | 0.00 | 0.00 |
| 19 | A:CYS 481 |  | 59.48 | 0.00 | 0.00 |
| 20 | A:SER 482 |  | 72.79 | 0.00 | 0.00 |
| 21 | A:GLN 483 |  | 70.87 | 0.00 | 0.00 |
| 22 | A:TYR 484 |  | 4.11 | 0.00 | 0.00 |
| 23 | A:HIS 485 |  | 63.87 | 0.00 | 0.00 |
| 24 | A:LYS 486 |  | 126.63 | 0.00 | 0.00 |
| 25 | A:MET 487 |  | 105.46 | 0.00 | 0.00 |
| 26 | A:TYR 488 |  | 11.93 | 0.00 | 0.00 |
| 27 | A:ARG 489 |  | 136.57 | 0.00 | 0.00 |
| 28 | A:THR 490 |  | 72.95 | 0.00 | 0.00 |
| 29 | A:VAL 491 |  | 50.46 | 0.00 | 0.00 |
| 30 | A:LYS 492 |  | 57.75 | 0.00 | 0.00 |
| 31 | A:ALA 493 |  | 86.69 | 0.00 | 0.00 |
| 32 | A:ILE 494 |  | 129.29 | 0.00 | 0.00 |
| 33 | A:THR 495 |  | 103.82 | 0.00 | 0.00 |
| 34 | A:GLY 496 |  | 45.13 | 0.00 | 0.00 |
| 35 | A:ARG 497 |  | 162.70 | 0.00 | 0.00 |
| 36 | A:GLN 498 |  | 93.05 | 0.00 | 0.00 |
| 37 | A:ILE 499 |  | 66.63 | 0.00 | 0.00 |
| 38 | A:PHE 500 |  | 49.22 | 0.00 | 0.00 |
| 39 | A:GLN 501 |  | 42.00 | 0.00 | 0.00 |
| 40 | A:PRO 502 |  | 64.95 | 0.00 | 0.00 |
| 41 | A:LEU 503 |  | 63.66 | 0.00 | 0.00 |
| 42 | A:HIS 504 |  | 118.75 | 0.00 | 0.00 |
| 43 | A:ALA 505 |  | 36.23 | 0.00 | 0.00 |
| 44 | A:LEU 506 |  | 2.48 | 0.00 | 0.00 |
| 45 | A:ARG 507 |  | 164.79 | 0.00 | 0.00 |
| 46 | A:ASN 508 |  | 76.05 | 0.00 | 0.00 |
| 47 | A:ALA 509 |  | 24.96 | 0.00 | 0.00 |
| 48 | A:GLU 510 |  | 12.45 | 0.00 | 0.00 |
| 49 | A:LYS 511 |  | 104.68 | 0.00 | 0.00 |
| 50 | A:VAL 512 |  | 64.53 | 0.00 | 0.00 |
| 51 | A:LEU 513 |  | 28.32 | 0.00 | 0.00 |
| 52 | A:LEU 514 |  | 26.17 | 0.00 | 0.00 |
| 53 | A:PRO 515 |  | 8.77 | 0.00 | 0.00 |
| 54 | A:GLY 516 |  | 25.37 | 0.00 | 0.00 |
| 55 | A:TYR 517 |  | 114.29 | 0.00 | 0.00 |
| 56 | A:HIS 518 |  | 52.86 | 0.00 | 0.00 |
| 57 | A:PRO 519 |  | 106.53 | 0.00 | 0.00 |
| 58 | A:PHE 520 |  | 22.69 | 0.00 | 0.00 |
| 59 | A:GLU 521 |  | 92.78 | 0.00 | 0.00 |
| 60 | A:TRP 522 |  | 20.03 | 0.00 | 0.00 |
| 61 | A:GLN 523 |  | 77.71 | 0.00 | 0.00 |
| 62 | A:PRO 524 |  | 85.03 | 0.00 | 0.00 |
| 63 | A:PRO 525 |  | 114.82 | 0.00 | 0.00 |
| 64 | A:LEU 526 |  | 21.20 | 0.00 | 0.00 |
| 65 | A:LYS 527 |  | 160.37 | 0.99  \| | -0.01 |
| 66 | A:ASN 528 | H | 144.77 | 78.36  \|\|\|\|\|\| | -0.13 |
| 67 | A:VAL 529 |  | 33.22 | 17.13  \|\|\|\|\|\| | 0.27 |
| 68 | A:SER 530 |  | 78.31 | 39.33  \|\|\|\|\|\| | 0.40 |
| 69 | A:SER 531 |  | 108.37 | 0.00 | 0.00 |
| 70 | A:SER 532 |  | 59.67 | 3.58  \| | -0.03 |
| 71 | A:THR 533 |  | 36.04 | 0.00 | 0.00 |
| 72 | A:ASP 534 |  | 105.39 | 0.00 | 0.00 |
| 73 | A:VAL 535 |  | 34.76 | 0.67  \| | 0.01 |
| 74 | A:GLY 536 |  | 26.61 | 0.00 | 0.00 |
| 75 | A:ILE 537 |  | 61.01 | 0.00 | 0.00 |
| 76 | A:ILE 538 |  | 37.94 | 8.88  \|\|\| | 0.14 |
| 77 | A:ASP 539 |  | 57.51 | 0.00 | 0.00 |
| 78 | A:GLY 540 | H | 0.37 | 0.37  \|\|\|\|\|\|\|\|\|\|\| | -0.00 |
| 79 | A:LEU 541 | H | 33.04 | 3.24  \| | -0.03 |
| 80 | A:SER 542 | H | 59.65 | 53.33  \|\|\|\|\|\|\|\|\| | 0.14 |
| 81 | A:GLY 543 | H | 73.12 | 36.78  \|\|\|\|\|\| | 0.04 |
| 82 | A:LEU 544 |  | 71.53 | 20.92  \|\|\| | 0.33 |
| 83 | A:SER 545 | H | 87.18 | 67.31  \|\|\|\|\|\|\|\| | 0.32 |
| 84 | A:SER 546 |  | 90.77 | 2.33  \| | 0.01 |
| 85 | A:SER 547 |  | 62.33 | 54.69  \|\|\|\|\|\|\|\|\| | 0.33 |
| 86 | A:VAL 548 |  | 144.88 | 93.65  \|\|\|\|\|\|\| | 1.37 |
| 87 | A:ASP 549 | H | 145.00 | 113.55  \|\|\|\|\|\|\|\| | -0.74 |
| 88 | A:ASP 550 | HS | 55.22 | 50.29  \|\|\|\|\|\|\|\|\|\| | 0.59 |
| 89 | A:TYR 551 |  | 108.70 | 78.57  \|\|\|\|\|\|\|\| | 0.99 |
| 90 | A:PRO 552 |  | 81.19 | 51.10  \|\|\|\|\|\|\| | 0.82 |
| 91 | A:VAL 553 |  | 37.20 | 0.00 | 0.00 |
| 92 | A:ASP 554 |  | 42.54 | 0.00 | 0.00 |
| 93 | A:THR 555 |  | 5.86 | 0.00 | 0.00 |
| 94 | A:ILE 556 |  | 17.74 | 0.00 | 0.00 |
| 95 | A:ALA 557 |  | 0.00 | 0.00 | 0.00 |
| 96 | A:LYS 558 |  | 17.86 | 0.00 | 0.00 |
| 97 | A:ARG 559 | H | 11.94 | 11.79  \|\|\|\|\|\|\|\|\|\| | -0.59 |
| 98 | A:PHE 560 |  | 2.68 | 0.00 | 0.00 |
| 99 | A:ARG 561 | S | 74.67 | 45.95  \|\|\|\|\|\|\| | -0.21 |
| 100 | A:TYR 562 |  | 3.50 | 0.00 | 0.00 |
| 101 | A:ASP 563 |  | 10.76 | 0.00 | 0.00 |
| 102 | A:SER 564 |  | 9.42 | 0.00 | 0.00 |
| 103 | A:ALA 565 |  | 0.00 | 0.00 | 0.00 |
| 104 | A:LEU 566 |  | 1.17 | 0.00 | 0.00 |
| 105 | A:VAL 567 |  | 2.50 | 0.00 | 0.00 |
| 106 | A:SER 568 |  | 16.05 | 0.00 | 0.00 |
| 107 | A:ALA 569 |  | 0.58 | 0.00 | 0.00 |
| 108 | A:LEU 570 |  | 1.73 | 0.00 | 0.00 |
| 109 | A:MET 571 |  | 35.52 | 0.00 | 0.00 |
| 110 | A:ASP 572 |  | 23.55 | 0.00 | 0.00 |
| 111 | A:MET 573 |  | 4.33 | 0.00 | 0.00 |
| 112 | A:GLU 574 |  | 49.59 | 0.00 | 0.00 |
| 113 | A:GLU 575 |  | 85.47 | 0.00 | 0.00 |
| 114 | A:ASP 576 |  | 33.59 | 0.00 | 0.00 |
| 115 | A:ILE 577 |  | 1.18 | 0.00 | 0.00 |
| 116 | A:LEU 578 |  | 57.47 | 0.00 | 0.00 |
| 117 | A:GLU 579 |  | 82.90 | 0.00 | 0.00 |
| 118 | A:GLY 580 |  | 13.93 | 0.00 | 0.00 |
| 119 | A:MET 581 |  | 29.02 | 0.00 | 0.00 |
| 120 | A:ARG 582 |  | 158.45 | 0.00 | 0.00 |
| 121 | A:ALA 583 |  | 71.27 | 0.00 | 0.00 |
| 122 | A:GLN 584 |  | 77.80 | 0.00 | 0.00 |
| 123 | A:ASP 585 |  | 119.15 | 0.00 | 0.00 |
| 124 | A:LEU 586 |  | 27.09 | 0.00 | 0.00 |
| 125 | A:ASP 587 |  | 105.15 | 0.00 | 0.00 |
| 126 | A:ASP 588 |  | 107.15 | 0.00 | 0.00 |
| 127 | A:TYR 589 |  | 153.28 | 0.00 | 0.00 |
| 128 | A:LEU 590 |  | 0.00 | 0.00 | 0.00 |
| 129 | A:ASN 591 |  | 18.82 | 0.00 | 0.00 |
| 130 | A:GLY 592 |  | 24.87 | 0.00 | 0.00 |
| 131 | A:PRO 593 |  | 63.18 | 0.00 | 0.00 |
| 132 | A:PHE 594 |  | 0.27 | 0.00 | 0.00 |
| 133 | A:THR 595 |  | 33.55 | 0.00 | 0.00 |
| 134 | A:VAL 596 |  | 0.00 | 0.00 | 0.00 |
| 135 | A:VAL 597 |  | 34.32 | 0.00 | 0.00 |
| 136 | A:VAL 598 |  | 0.00 | 0.00 | 0.00 |
| 137 | A:LYS 599 |  | 9.90 | 0.00 | 0.00 |
| 138 | A:GLU 600 |  | 7.59 | 0.00 | 0.00 |
| 139 | A:SER 601 |  | 9.43 | 0.00 | 0.00 |
| 140 | A:CYS 602 |  | 6.79 | 0.00 | 0.00 |
| 141 | A:ASP 603 |  | 24.51 | 0.00 | 0.00 |
| 142 | A:GLY 604 |  | 17.69 | 0.00 | 0.00 |
| 143 | A:MET 605 |  | 37.01 | 0.00 | 0.00 |
| 144 | A:GLY 606 |  | 40.30 | 0.00 | 0.00 |
| 145 | A:ASP 607 |  | 142.35 | 26.07  \|\| | -0.18 |
| 146 | A:VAL 608 |  | 15.69 | 0.00 | 0.00 |
| 147 | A:SER 609 |  | 99.10 | 32.34  \|\|\|\| | 0.18 |
| 148 | A:GLU 610 |  | 63.76 | 7.66  \|\| | -0.06 |
| 149 | A:LYS 611 |  | 48.84 | 0.00 | 0.00 |
| 150 | A:HIS 612 |  | 183.20 | 0.00 | 0.00 |
| 151 | A:GLY 613 |  | 41.58 | 0.00 | 0.00 |
| 152 | A:SER 614 |  | 95.24 | 0.00 | 0.00 |
| 153 | A:GLY 615 |  | 19.59 | 0.00 | 0.00 |
| 154 | A:PRO 616 |  | 61.10 | 4.36  \| | 0.07 |
| 155 | A:VAL 617 |  | 134.47 | 69.25  \|\|\|\|\|\| | 1.11 |
| 156 | A:VAL 618 |  | 6.12 | 0.00 | 0.00 |
| 157 | A:PRO 619 |  | 47.60 | 0.00 | 0.00 |
| 158 | A:GLU 620 |  | 101.84 | 79.26  \|\|\|\|\|\|\|\| | -0.66 |
| 159 | A:LYS 621 |  | 76.13 | 0.33  \| | 0.01 |
| 160 | A:ALA 622 |  | 0.31 | 0.00 | 0.00 |
| 161 | A:VAL 623 |  | 3.69 | 0.00 | 0.00 |
| 162 | A:ARG 624 |  | 1.68 | 0.00 | 0.00 |
| 163 | A:PHE 625 |  | 19.00 | 0.00 | 0.00 |
| 164 | A:SER 626 |  | 5.59 | 0.00 | 0.00 |
| 165 | A:PHE 627 |  | 2.22 | 0.00 | 0.00 |
| 166 | A:THR 628 |  | 8.40 | 0.00 | 0.00 |
| 167 | A:VAL 629 |  | 8.26 | 0.00 | 0.00 |
| 168 | A:MET 630 |  | 9.38 | 0.00 | 0.00 |
| 169 | A:LYS 631 |  | 89.00 | 0.00 | 0.00 |
| 170 | A:ILE 632 |  | 0.83 | 0.00 | 0.00 |
| 171 | A:THR 633 |  | 23.44 | 0.00 | 0.00 |
| 172 | A:ILE 634 |  | 1.43 | 0.00 | 0.00 |
| 173 | A:ALA 635 |  | 39.19 | 0.00 | 0.00 |
| 174 | A:HIS 636 |  | 79.95 | 0.00 | 0.00 |
| 175 | A:GLY 637 |  | 68.80 | 0.00 | 0.00 |
| 176 | A:SER 638 |  | 120.61 | 0.00 | 0.00 |
| 177 | A:GLN 639 |  | 125.02 | 0.00 | 0.00 |
| 178 | A:ASN 640 |  | 91.81 | 0.00 | 0.00 |
| 179 | A:VAL 641 |  | 39.34 | 0.00 | 0.00 |
| 180 | A:LYS 642 |  | 95.85 | 0.00 | 0.00 |
| 181 | A:VAL 643 |  | 14.54 | 0.00 | 0.00 |
| 182 | A:PHE 644 |  | 30.44 | 0.00 | 0.00 |
| 183 | A:GLU 645 |  | 69.18 | 0.00 | 0.00 |
| 184 | A:GLU 646 |  | 24.65 | 0.00 | 0.00 |
| 185 | A:ALA 647 |  | 95.04 | 0.00 | 0.00 |
| 186 | A:LYS 648 |  | 143.56 | 0.00 | 0.00 |
| 187 | A:PRO 649 |  | 17.20 | 0.00 | 0.00 |
| 188 | A:ASN 650 |  | 42.21 | 0.00 | 0.00 |
| 189 | A:SER 651 |  | 28.28 | 0.00 | 0.00 |
| 190 | A:GLU 652 |  | 38.62 | 0.00 | 0.00 |
| 191 | A:LEU 653 |  | 65.84 | 0.00 | 0.00 |
| 192 | A:CYS 654 |  | 15.14 | 0.00 | 0.00 |
| 193 | A:CYS 655 |  | 19.44 | 0.00 | 0.00 |
| 194 | A:LYS 656 |  | 15.16 | 0.00 | 0.00 |
| 195 | A:PRO 657 |  | 5.99 | 0.00 | 0.00 |
| 196 | A:LEU 658 |  | 0.00 | 0.00 | 0.00 |
| 197 | A:CYS 659 |  | 0.74 | 0.00 | 0.00 |
| 198 | A:LEU 660 |  | 0.50 | 0.00 | 0.00 |
| 199 | A:MET 661 |  | 0.00 | 0.00 | 0.00 |
| 200 | A:LEU 662 |  | 26.44 | 0.00 | 0.00 |
| 201 | A:ALA 663 |  | 5.29 | 0.00 | 0.00 |
| 202 | A:ASP 664 |  | 41.10 | 2.93  \| | -0.02 |
| 203 | A:GLU 665 |  | 33.26 | 0.00 | 0.00 |
| 204 | A:SER 666 |  | 75.70 | 0.49  \| | -0.01 |
| 205 | A:ASP 667 | S | 52.89 | 40.61  \|\|\|\|\|\|\|\| | 0.09 |
| 206 | A:HIS 668 |  | 125.66 | 81.34  \|\|\|\|\|\|\| | 0.96 |
| 207 | A:GLU 669 | HS | 96.59 | 92.71  \|\|\|\|\|\|\|\|\|\| | -0.23 |
| 208 | A:THR 670 |  | 8.37 | 0.00 | 0.00 |
| 209 | A:LEU 671 |  | 21.38 | 0.00 | 0.00 |
| 210 | A:THR 672 | H | 50.28 | 31.78  \|\|\|\|\|\|\| | 0.12 |
| 211 | A:ALA 673 | H | 46.85 | 33.19  \|\|\|\|\|\|\|\| | 0.27 |
| 212 | A:ILE 674 |  | 1.35 | 0.33  \|\|\| | 0.01 |
| 213 | A:LEU 675 |  | 16.67 | 0.00 | 0.00 |
| 214 | A:SER 676 | H | 55.68 | 40.29  \|\|\|\|\|\|\|\| | -0.02 |
| 215 | A:PRO 677 |  | 34.61 | 34.61  \|\|\|\|\|\|\|\|\|\| | 0.54 |
| 216 | A:LEU 678 |  | 6.99 | 0.00 | 0.00 |
| 217 | A:ILE 679 |  | 35.49 | 0.00 | 0.00 |
| 218 | A:ALA 680 |  | 53.22 | 20.43  \|\|\|\| | 0.33 |
| 219 | A:GLU 681 |  | 11.95 | 10.08  \|\|\|\|\|\|\|\|\| | 0.10 |
| 220 | A:ARG 682 |  | 12.19 | 0.00 | 0.00 |
| 221 | A:GLU 683 |  | 93.70 | 0.00 | 0.00 |
| 222 | A:ALA 684 |  | 12.08 | 0.00 | 0.00 |
| 223 | A:MET 685 |  | 1.27 | 0.00 | 0.00 |
| 224 | A:LYS 686 |  | 92.28 | 0.00 | 0.00 |
| 225 | A:SER 687 |  | 87.62 | 0.00 | 0.00 |
| 226 | A:SER 688 |  | 4.97 | 0.00 | 0.00 |
| 227 | A:GLN 689 |  | 71.35 | 0.00 | 0.00 |
| 228 | A:LEU 690 |  | 0.00 | 0.00 | 0.00 |
| 229 | A:MET 691 |  | 47.00 | 0.00 | 0.00 |
| 230 | A:LEU 692 |  | 3.19 | 0.00 | 0.00 |
| 231 | A:GLU 693 |  | 98.22 | 0.00 | 0.00 |
| 232 | A:MET 694 |  | 1.02 | 0.00 | 0.00 |
| 233 | A:GLY 695 |  | 31.40 | 0.00 | 0.00 |
| 234 | A:GLY 696 |  | 63.50 | 0.00 | 0.00 |
| 235 | A:ILE 697 |  | 79.84 | 0.00 | 0.00 |
| 236 | A:LEU 698 |  | 113.19 | 0.00 | 0.00 |
| 237 | A:ARG 699 |  | 9.21 | 0.00 | 0.00 |
| 238 | A:THR 700 |  | 36.41 | 0.00 | 0.00 |
| 239 | A:PHE 701 |  | 1.25 | 0.00 | 0.00 |
| 240 | A:LYS 702 |  | 99.00 | 0.00 | 0.00 |
| 241 | A:PHE 703 |  | 13.01 | 0.00 | 0.00 |
| 242 | A:ILE 704 |  | 69.98 | 0.00 | 0.00 |
| 243 | A:PHE 705 |  | 9.13 | 0.00 | 0.00 |
| 244 | A:ARG 706 |  | 92.74 | 0.00 | 0.00 |
| 245 | A:GLY 707 |  | 6.75 | 0.00 | 0.00 |
| 246 | A:THR 708 |  | 9.25 | 0.00 | 0.00 |
| 247 | A:GLY 709 |  | 1.93 | 0.00 | 0.00 |
| 248 | A:TYR 710 |  | 24.36 | 0.00 | 0.00 |
| 249 | A:ASP 711 |  | 26.61 | 0.00 | 0.00 |
| 250 | A:GLU 712 |  | 17.70 | 0.00 | 0.00 |
| 251 | A:LYS 713 |  | 100.44 | 0.00 | 0.00 |
| 252 | A:LEU 714 |  | 11.39 | 0.00 | 0.00 |
| 253 | A:VAL 715 |  | 22.93 | 0.00 | 0.00 |
| 254 | A:ARG 716 |  | 2.97 | 0.00 | 0.00 |
| 255 | A:GLU 717 |  | 59.16 | 0.00 | 0.00 |
| 256 | A:VAL 718 |  | 16.49 | 0.00 | 0.00 |
| 257 | A:GLU 719 |  | 9.62 | 0.00 | 0.00 |
| 258 | A:GLY 720 |  | 3.27 | 0.00 | 0.00 |
| 259 | A:LEU 721 |  | 18.98 | 0.00 | 0.00 |
| 260 | A:GLU 722 | HS | 53.61 | 29.17  \|\|\|\|\|\| | -0.03 |
| 261 | A:ALA 723 |  | 66.22 | 6.94  \|\| | -0.08 |
| 262 | A:SER 724 |  | 54.70 | 0.00 | 0.00 |
| 263 | A:GLY 725 |  | 68.07 | 0.00 | 0.00 |
| 264 | A:SER 726 |  | 20.70 | 7.95  \|\|\|\| | 0.07 |
| 265 | A:VAL 727 |  | 89.21 | 0.00 | 0.00 |
| 266 | A:TYR 728 |  | 24.23 | 0.00 | 0.00 |
| 267 | A:ILE 729 |  | 0.00 | 0.00 | 0.00 |
| 268 | A:CYS 730 |  | 2.68 | 0.00 | 0.00 |
| 269 | A:THR 731 |  | 14.63 | 0.00 | 0.00 |
| 270 | A:LEU 732 |  | 5.01 | 0.00 | 0.00 |
| 271 | A:CYS 733 |  | 2.21 | 0.00 | 0.00 |
| 272 | A:ASP 734 |  | 49.85 | 0.00 | 0.00 |
| 273 | A:ALA 735 |  | 9.41 | 0.00 | 0.00 |
| 274 | A:THR 736 |  | 33.82 | 0.00 | 0.00 |
| 275 | A:ARG 737 |  | 57.23 | 0.00 | 0.00 |
| 276 | A:LEU 738 |  | 123.85 | 0.00 | 0.00 |
| 277 | A:GLU 739 |  | 101.85 | 0.00 | 0.00 |
| 278 | A:ALA 740 |  | 0.38 | 0.00 | 0.00 |
| 279 | A:SER 741 |  | 18.64 | 0.00 | 0.00 |
| 280 | A:GLN 742 |  | 163.72 | 0.00 | 0.00 |
| 281 | A:ASN 743 |  | 61.37 | 0.00 | 0.00 |
| 282 | A:LEU 744 |  | 16.26 | 0.00 | 0.00 |
| 283 | A:VAL 745 |  | 26.36 | 0.00 | 0.00 |
| 284 | A:PHE 746 |  | 132.43 | 0.00 | 0.00 |
| 285 | A:HIS 747 |  | 43.57 | 0.00 | 0.00 |
| 286 | A:SER 748 |  | 84.21 | 0.00 | 0.00 |
| 287 | A:ILE 749 |  | 21.68 | 0.00 | 0.00 |
| 288 | A:THR 750 |  | 71.36 | 0.00 | 0.00 |
| 289 | A:ARG 751 |  | 8.56 | 0.00 | 0.00 |
| 290 | A:SER 752 |  | 53.78 | 0.00 | 0.00 |
| 291 | A:HIS 753 |  | 40.39 | 0.00 | 0.00 |
| 292 | A:ALA 754 |  | 64.48 | 0.00 | 0.00 |
| 293 | A:GLU 755 |  | 61.39 | 0.00 | 0.00 |
| 294 | A:ASN 756 |  | 5.96 | 0.00 | 0.00 |
| 295 | A:LEU 757 |  | 82.23 | 0.00 | 0.00 |
| 296 | A:GLU 758 |  | 115.12 | 0.00 | 0.00 |
| 297 | A:ARG 759 |  | 35.33 | 0.00 | 0.00 |
| 298 | A:TYR 760 |  | 40.54 | 13.87  \|\|\|\| | -0.16 |
| 299 | A:GLU 761 |  | 80.56 | 0.00 | 0.00 |
| 300 | A:VAL 762 |  | 43.69 | 0.00 | 0.00 |
| 301 | A:TRP 763 |  | 85.25 | 19.53  \|\|\| | 0.25 |
| 302 | A:ARG 764 |  | 150.20 | 36.59  \|\|\| | 0.11 |
| 303 | A:SER 765 |  | 59.57 | 0.00 | 0.00 |
| 304 | A:ASN 766 |  | 37.16 | 0.33  \| | 0.01 |
| 305 | A:PRO 767 |  | 71.06 | 0.00 | 0.00 |
| 306 | A:TYR 768 |  | 143.93 | 0.00 | 0.00 |
| 307 | A:HIS 769 |  | 161.63 | 0.00 | 0.00 |
| 308 | A:GLU 770 |  | 51.61 | 0.00 | 0.00 |
| 309 | A:THR 771 |  | 89.43 | 0.00 | 0.00 |
| 310 | A:VAL 772 |  | 109.86 | 5.52  \| | 0.09 |
| 311 | A:ASP 773 |  | 100.87 | 0.00 | 0.00 |
| 312 | A:GLU 774 |  | 91.17 | 0.00 | 0.00 |
| 313 | A:LEU 775 |  | 14.73 | 0.00 | 0.00 |
| 314 | A:ARG 776 |  | 59.25 | 23.49  \|\|\|\| | 0.81 |
| 315 | A:ASP 777 |  | 91.02 | 0.00 | 0.00 |
| 316 | A:ARG 778 |  | 113.40 | 0.00 | 0.00 |
| 317 | A:VAL 779 |  | 2.67 | 0.00 | 0.00 |
| 318 | A:LYS 780 |  | 50.74 | 0.00 | 0.00 |
| 319 | A:GLY 781 |  | 4.42 | 0.00 | 0.00 |
| 320 | A:VAL 782 |  | 0.00 | 0.00 | 0.00 |
| 321 | A:SER 783 |  | 27.21 | 0.12  \| | -0.00 |
| 322 | A:ALA 784 |  | 44.02 | 16.57  \|\|\|\| | 0.27 |
| 323 | A:LYS 785 |  | 104.48 | 21.02  \|\|\| | -0.43 |
| 324 | A:PRO 786 |  | 5.53 | 0.00 | 0.00 |
| 325 | A:PHE 787 |  | 92.65 | 55.30  \|\|\|\|\|\| | 0.88 |
| 326 | A:ILE 788 |  | 34.07 | 0.00 | 0.00 |
| 327 | A:GLU 789 |  | 64.23 | 0.00 | 0.00 |
| 328 | A:THR 790 |  | 18.28 | 0.00 | 0.00 |
| 329 | A:VAL 791 |  | 15.94 | 0.00 | 0.00 |
| 330 | A:PRO 792 |  | 57.81 | 0.00 | 0.00 |
| 331 | A:SER 793 |  | 17.87 | 0.00 | 0.00 |
| 332 | A:ILE 794 |  | 11.31 | 0.00 | 0.00 |
| 333 | A:ASP 795 |  | 8.49 | 0.00 | 0.00 |
| 334 | A:ALA 796 |  | 3.63 | 0.00 | 0.00 |
| 335 | A:LEU 797 |  | 37.81 | 0.00 | 0.00 |
| 336 | A:HIS 798 |  | 57.21 | 0.00 | 0.00 |
| 337 | A:CYS 799 |  | 1.84 | 0.00 | 0.00 |
| 338 | A:ASP 800 |  | 13.50 | 0.00 | 0.00 |
| 339 | A:ILE 801 |  | 37.08 | 0.00 | 0.00 |
| 340 | A:GLY 802 |  | 21.20 | 0.00 | 0.00 |
| 341 | A:ASN 803 |  | 2.30 | 0.00 | 0.00 |
| 342 | A:ALA 804 |  | 0.00 | 0.00 | 0.00 |
| 343 | A:ALA 805 |  | 32.98 | 0.00 | 0.00 |
| 344 | A:GLU 806 |  | 7.07 | 0.00 | 0.00 |
| 345 | A:PHE 807 |  | 0.94 | 0.00 | 0.00 |
| 346 | A:TYR 808 |  | 49.39 | 0.00 | 0.00 |
| 347 | A:LYS 809 |  | 105.52 | 0.00 | 0.00 |
| 348 | A:ILE 810 |  | 0.17 | 0.00 | 0.00 |
| 349 | A:PHE 811 |  | 1.72 | 0.00 | 0.00 |
| 350 | A:GLN 812 |  | 33.65 | 0.00 | 0.00 |
| 351 | A:LEU 813 |  | 54.31 | 0.00 | 0.00 |
| 352 | A:GLU 814 |  | 0.61 | 0.00 | 0.00 |
| 353 | A:ILE 815 |  | 24.80 | 0.00 | 0.00 |
| 354 | A:GLY 816 |  | 9.92 | 0.00 | 0.00 |
| 355 | A:GLU 817 |  | 54.48 | 0.00 | 0.00 |
| 356 | A:ALA 818 |  | 29.13 | 0.00 | 0.00 |
| 357 | A:TYR 819 |  | 70.03 | 0.00 | 0.00 |
| 358 | A:LYS 820 |  | 137.58 | 0.00 | 0.00 |
| 359 | A:ASN 821 |  | 76.47 | 0.00 | 0.00 |
| 360 | A:PRO 822 |  | 122.83 | 0.00 | 0.00 |
| 361 | A:HIS 823 |  | 89.36 | 0.00 | 0.00 |
| 362 | A:ALA 824 |  | 94.94 | 0.00 | 0.00 |
| 363 | A:SER 825 |  | 38.45 | 0.00 | 0.00 |
| 364 | A:LYS 826 |  | 170.36 | 0.00 | 0.00 |
| 365 | A:GLU 827 |  | 111.74 | 0.00 | 0.00 |
| 366 | A:GLU 828 |  | 72.53 | 0.00 | 0.00 |
| 367 | A:ARG 829 |  | 118.39 | 0.00 | 0.00 |
| 368 | A:LYS 830 |  | 138.31 | 0.00 | 0.00 |
| 369 | A:ARG 831 |  | 154.36 | 0.00 | 0.00 |
| 370 | A:TRP 832 |  | 72.73 | 0.00 | 0.00 |
| 371 | A:GLN 833 |  | 85.28 | 0.00 | 0.00 |
| 372 | A:ALA 834 |  | 38.65 | 0.00 | 0.00 |
| 373 | A:THR 835 |  | 57.89 | 0.00 | 0.00 |
| 374 | A:LEU 836 |  | 0.85 | 0.00 | 0.00 |
| 375 | A:ASP 837 |  | 35.51 | 0.00 | 0.00 |
| 376 | A:LYS 838 |  | 145.20 | 0.00 | 0.00 |
| 377 | A:HIS 839 |  | 18.50 | 0.00 | 0.00 |
| 378 | A:LEU 840 |  | 5.51 | 0.00 | 0.00 |
| 379 | A:ARG 841 |  | 160.58 | 0.00 | 0.00 |
| 380 | A:LYS 842 |  | 161.98 | 0.00 | 0.00 |
| 381 | A:LYS 843 |  | 87.13 | 0.00 | 0.00 |
| 382 | A:MET 844 |  | 45.40 | 0.00 | 0.00 |
| 383 | A:ASN 845 |  | 127.39 | 0.00 | 0.00 |
| 384 | A:LEU 846 |  | 16.53 | 0.00 | 0.00 |
| 385 | A:LYS 847 |  | 154.88 | 0.00 | 0.00 |
| 386 | A:PRO 848 |  | 84.37 | 0.00 | 0.00 |
| 387 | A:ILE 849 |  | 62.47 | 0.00 | 0.00 |
| 388 | A:MET 850 |  | 176.37 | 0.00 | 0.00 |
| 389 | A:ARG 851 |  | 184.58 | 0.00 | 0.00 |
| 390 | A:MET 852 |  | 36.22 | 0.00 | 0.00 |
| 391 | A:ASN 853 |  | 76.02 | 0.00 | 0.00 |
| 392 | A:GLY 854 |  | 19.26 | 0.00 | 0.00 |
| 393 | A:ASN 855 |  | 88.37 | 0.00 | 0.00 |
| 394 | A:PHE 856 |  | 18.08 | 0.00 | 0.00 |
| 395 | A:ALA 857 |  | 0.00 | 0.00 | 0.00 |
| 396 | A:ARG 858 |  | 126.01 | 0.00 | 0.00 |
| 397 | A:LYS 859 |  | 103.16 | 0.00 | 0.00 |
| 398 | A:LEU 860 |  | 1.52 | 0.00 | 0.00 |
| 399 | A:MET 861 |  | 2.94 | 0.00 | 0.00 |
| 400 | A:THR 862 |  | 48.78 | 0.00 | 0.00 |
| 401 | A:LYS 863 |  | 74.36 | 0.00 | 0.00 |
| 402 | A:GLU 864 |  | 126.67 | 0.00 | 0.00 |
| 403 | A:THR 865 |  | 0.00 | 0.00 | 0.00 |
| 404 | A:VAL 866 |  | 0.50 | 0.00 | 0.00 |
| 405 | A:GLU 867 |  | 76.91 | 0.00 | 0.00 |
| 406 | A:ALA 868 |  | 3.78 | 0.00 | 0.00 |
| 407 | A:VAL 869 |  | 0.50 | 0.00 | 0.00 |
| 408 | A:CYS 870 |  | 5.32 | 0.00 | 0.00 |
| 409 | A:GLU 871 |  | 109.89 | 0.00 | 0.00 |
| 410 | A:LEU 872 |  | 52.23 | 0.00 | 0.00 |
| 411 | A:ILE 873 |  | 12.98 | 0.00 | 0.00 |
| 412 | A:PRO 874 |  | 90.32 | 0.00 | 0.00 |
| 413 | A:SER 875 |  | 61.62 | 0.00 | 0.00 |
| 414 | A:GLU 876 |  | 145.44 | 0.00 | 0.00 |
| 415 | A:GLU 877 |  | 113.51 | 0.00 | 0.00 |
| 416 | A:ARG 878 |  | 24.47 | 0.00 | 0.00 |
| 417 | A:HIS 879 |  | 50.83 | 0.00 | 0.00 |
| 418 | A:GLU 880 |  | 74.61 | 0.00 | 0.00 |
| 419 | A:ALA 881 |  | 3.57 | 0.00 | 0.00 |
| 420 | A:LEU 882 |  | 0.00 | 0.00 | 0.00 |
| 421 | A:ARG 883 |  | 75.06 | 0.00 | 0.00 |
| 422 | A:GLU 884 |  | 88.64 | 0.00 | 0.00 |
| 423 | A:LEU 885 |  | 0.17 | 0.00 | 0.00 |
| 424 | A:MET 886 |  | 1.01 | 0.00 | 0.00 |
| 425 | A:ASP 887 |  | 33.18 | 0.00 | 0.00 |
| 426 | A:LEU 888 |  | 20.09 | 0.00 | 0.00 |
| 427 | A:TYR 889 |  | 13.98 | 0.00 | 0.00 |
| 428 | A:LEU 890 |  | 59.05 | 0.00 | 0.00 |
| 429 | A:LYS 891 |  | 112.33 | 0.00 | 0.00 |
| 430 | A:MET 892 |  | 11.04 | 0.00 | 0.00 |
| 431 | A:LYS 893 |  | 21.04 | 0.00 | 0.00 |
| 432 | A:PRO 894 |  | 24.43 | 0.00 | 0.00 |
| 433 | A:VAL 895 |  | 0.00 | 0.00 | 0.00 |
| 434 | A:TRP 896 |  | 11.13 | 0.00 | 0.00 |
| 435 | A:ARG 897 |  | 132.22 | 0.00 | 0.00 |
| 436 | A:SER 898 |  | 10.62 | 0.00 | 0.00 |
| 437 | A:SER 899 |  | 64.58 | 0.00 | 0.00 |
| 438 | A:CYS 900 |  | 45.46 | 0.00 | 0.00 |
| 439 | A:PRO 901 |  | 0.17 | 0.00 | 0.00 |
| 440 | A:ALA 902 |  | 62.64 | 0.00 | 0.00 |
| 441 | A:LYS 903 |  | 160.24 | 0.00 | 0.00 |
| 442 | A:GLU 904 |  | 92.80 | 0.00 | 0.00 |
| 443 | A:CYS 905 |  | 46.07 | 0.00 | 0.00 |
| 444 | A:PRO 906 |  | 68.72 | 0.00 | 0.00 |
| 445 | A:GLU 907 |  | 98.50 | 0.00 | 0.00 |
| 446 | A:SER 908 |  | 23.14 | 0.00 | 0.00 |
| 447 | A:LEU 909 |  | 43.89 | 0.00 | 0.00 |
| 448 | A:CYS 910 |  | 95.92 | 0.00 | 0.00 |
| 449 | A:GLN 911 |  | 81.15 | 0.00 | 0.00 |
| 450 | A:TYR 912 |  | 4.31 | 0.00 | 0.00 |
| 451 | A:SER 913 |  | 59.84 | 0.00 | 0.00 |
| 452 | A:PHE 914 |  | 123.01 | 0.00 | 0.00 |
| 453 | A:ASN 915 |  | 11.54 | 0.00 | 0.00 |
| 454 | A:SER 916 |  | 3.56 | 0.00 | 0.00 |
| 455 | A:GLN 917 |  | 50.42 | 0.00 | 0.00 |
| 456 | A:ARG 918 |  | 72.50 | 0.00 | 0.00 |
| 457 | A:PHE 919 |  | 5.95 | 0.00 | 0.00 |
| 458 | A:ALA 920 |  | 7.02 | 0.00 | 0.00 |
| 459 | A:GLU 921 |  | 87.06 | 0.00 | 0.00 |
| 460 | A:LEU 922 |  | 19.07 | 0.00 | 0.00 |
| 461 | A:LEU 923 |  | 1.51 | 0.00 | 0.00 |
| 462 | A:SER 924 |  | 59.16 | 0.00 | 0.00 |
| 463 | A:THR 925 |  | 83.81 | 0.00 | 0.00 |
| 464 | A:LYS 926 |  | 83.84 | 0.00 | 0.00 |
| 465 | A:PHE 927 |  | 0.00 | 0.00 | 0.00 |
| 466 | A:LYS 928 |  | 138.88 | 0.00 | 0.00 |
| 467 | A:TYR 929 |  | 71.75 | 0.00 | 0.00 |
| 468 | A:ARG 930 |  | 50.17 | 0.00 | 0.00 |
| 469 | A:TYR 931 |  | 23.14 | 0.00 | 0.00 |
| 470 | A:GLU 932 |  | 151.96 | 0.00 | 0.00 |
| 471 | A:GLY 933 |  | 13.55 | 0.00 | 0.00 |
| 472 | A:LYS 934 |  | 150.27 | 0.00 | 0.00 |
| 473 | A:ILE 935 |  | 9.18 | 0.00 | 0.00 |
| 474 | A:THR 936 |  | 53.83 | 0.00 | 0.00 |
| 475 | A:ASN 937 |  | 19.88 | 0.00 | 0.00 |
| 476 | A:TYR 938 |  | 41.00 | 0.00 | 0.00 |
| 477 | A:PHE 939 |  | 3.75 | 0.00 | 0.00 |
| 478 | A:HIS 940 |  | 1.56 | 0.00 | 0.00 |
| 479 | A:LYS 941 |  | 10.23 | 0.00 | 0.00 |
| 480 | A:THR 942 |  | 18.29 | 0.00 | 0.00 |
| 481 | A:LEU 943 |  | 8.18 | 0.00 | 0.00 |
| 482 | A:ALA 944 |  | 12.14 | 0.00 | 0.00 |
| 483 | A:HIS 945 |  | 1.90 | 0.00 | 0.00 |
| 484 | A:VAL 946 |  | 0.67 | 0.00 | 0.00 |
| 485 | A:PRO 947 |  | 39.64 | 0.00 | 0.00 |
| 486 | A:GLU 948 |  | 68.62 | 0.00 | 0.00 |
| 487 | A:ILE 949 |  | 1.50 | 0.00 | 0.00 |
| 488 | A:ILE 950 |  | 8.25 | 0.00 | 0.00 |
| 489 | A:GLU 951 |  | 123.06 | 0.00 | 0.00 |
| 490 | A:ARG 952 |  | 132.44 | 0.00 | 0.00 |
| 491 | A:ASP 953 |  | 45.38 | 0.00 | 0.00 |
| 492 | A:GLY 954 |  | 38.92 | 0.00 | 0.00 |
| 493 | A:SER 955 |  | 10.88 | 0.00 | 0.00 |
| 494 | A:ILE 956 |  | 1.21 | 0.00 | 0.00 |
| 495 | A:GLY 957 |  | 4.82 | 0.00 | 0.00 |
| 496 | A:ALA 958 |  | 9.36 | 0.00 | 0.00 |
| 497 | A:TRP 959 |  | 31.31 | 0.00 | 0.00 |
| 498 | A:ALA 960 |  | 4.66 | 0.00 | 0.00 |
| 499 | A:SER 961 |  | 7.90 | 0.00 | 0.00 |
| 500 | A:GLU 962 |  | 63.61 | 0.00 | 0.00 |
| 501 | A:GLY 963 |  | 5.58 | 0.00 | 0.00 |
| 502 | A:ASN 964 |  | 7.98 | 0.00 | 0.00 |
| 503 | A:GLU 965 |  | 89.28 | 0.00 | 0.00 |
| 504 | A:SER 966 |  | 37.55 | 0.00 | 0.00 |
| 505 | A:GLY 967 |  | 10.83 | 0.00 | 0.00 |
| 506 | A:ASN 968 |  | 8.16 | 0.00 | 0.00 |
| 507 | A:LYS 969 |  | 110.87 | 0.00 | 0.00 |
| 508 | A:LEU 970 |  | 8.87 | 0.00 | 0.00 |
| 509 | A:PHE 971 |  | 12.66 | 0.00 | 0.00 |
| 510 | A:ARG 972 |  | 106.00 | 0.00 | 0.00 |
| 511 | A:ARG 973 |  | 76.30 | 0.00 | 0.00 |
| 512 | A:PHE 974 |  | 6.86 | 0.00 | 0.00 |
| 513 | A:ARG 975 |  | 22.32 | 0.00 | 0.00 |
| 514 | A:LYS 976 |  | 127.09 | 0.00 | 0.00 |
| 515 | A:MET 977 |  | 107.52 | 0.00 | 0.00 |
| 516 | A:ASN 978 |  | 18.29 | 0.00 | 0.00 |
| 517 | A:ALA 979 |  | 20.39 | 0.00 | 0.00 |
| 518 | A:ARG 980 |  | 113.87 | 0.00 | 0.00 |
| 519 | A:GLN 981 |  | 118.36 | 0.00 | 0.00 |
| 520 | A:SER 982 |  | 34.31 | 0.00 | 0.00 |
| 521 | A:LYS 983 |  | 17.81 | 0.00 | 0.00 |
| 522 | A:TYR 984 |  | 122.05 | 0.00 | 0.00 |
| 523 | A:TYR 985 |  | 92.60 | 0.00 | 0.00 |
| 524 | A:GLU 986 |  | 24.02 | 0.00 | 0.00 |
| 525 | A:MET 987 |  | 3.51 | 0.00 | 0.00 |
| 526 | A:GLU 988 |  | 56.75 | 0.00 | 0.00 |
| 527 | A:ASP 989 |  | 17.06 | 0.00 | 0.00 |
| 528 | A:VAL 990 |  | 0.00 | 0.00 | 0.00 |
| 529 | A:LEU 991 |  | 0.17 | 0.00 | 0.00 |
| 530 | A:LYS 992 |  | 50.39 | 0.00 | 0.00 |
| 531 | A:HIS 993 |  | 30.74 | 0.00 | 0.00 |
| 532 | A:HIS 994 |  | 6.49 | 0.00 | 0.00 |
| 533 | A:TRP 995 |  | 3.61 | 0.00 | 0.00 |
| 534 | A:LEU 996 |  | 0.95 | 0.00 | 0.00 |
| 535 | A:TYR 997 |  | 21.67 | 0.00 | 0.00 |
| 536 | A:THR 998 |  | 4.27 | 0.00 | 0.00 |
| 537 | A:SER 999 |  | 3.62 | 0.00 | 0.00 |
| 538 | A:LYS1000 |  | 32.98 | 0.00 | 0.00 |
| 539 | A:TYR1001 |  | 74.44 | 0.00 | 0.00 |
| 540 | A:LEU1002 |  | 1.67 | 0.00 | 0.00 |
| 541 | A:GLN1003 |  | 30.50 | 0.00 | 0.00 |
| 542 | A:LYS1004 |  | 62.29 | 0.00 | 0.00 |
| 543 | A:PHE1005 |  | 33.81 | 0.00 | 0.00 |
| 544 | A:MET1006 |  | 57.73 | 0.00 | 0.00 |
| 545 | A:ASN1007 |  | 119.03 | 0.00 | 0.00 |
| 546 | A:ALA1008 |  | 84.51 | 0.00 | 0.00 |
| 547 | A:HIS1009 |  | 125.71 | 0.00 | 0.00 |
| 548 | A:LYS1010 |  | 197.15 | 0.00 | 0.00 |

**Supplementary Table S3.** Interfacing residues of RAG2 in RAG1-RAG2.

| **Num** | [**Structure 2**](javascript:openWindow('pi_ipage_res2.html',400,250);) | [**HSDC**](javascript:openWindow('pi_ipage_hs.html',400,250);) | [**ASA**](javascript:openWindow('pi_ipage_asa.html',400,250);) | [**BSA**](javascript:openWindow('pi_ipage_bsa.html',400,250);) | [**Δ**^i^**G**](javascript:openWindow('pi_ipage_rdg.html',400,250);) |
| --- | --- | --- | --- | --- | --- |
| 1 | D:MET   1 |  | 66.46 | 0.00 | 0.00 |
| 2 | D:SER   2 |  | 80.61 | 0.00 | 0.00 |
| 3 | D:LEU   3 |  | 37.15 | 0.00 | 0.00 |
| 4 | D:GLN   4 |  | 104.06 | 0.00 | 0.00 |
| 5 | D:MET   5 |  | 100.92 | 0.00 | 0.00 |
| 6 | D:ILE   6 |  | 12.50 | 0.00 | 0.00 |
| 7 | D:THR   7 |  | 62.36 | 0.00 | 0.00 |
| 8 | D:VAL   8 |  | 4.07 | 0.00 | 0.00 |
| 9 | D:GLY   9 |  | 55.25 | 0.00 | 0.00 |
| 10 | D:ASN  10 |  | 122.91 | 0.00 | 0.00 |
| 11 | D:ASN  11 |  | 42.12 | 0.00 | 0.00 |
| 12 | D:MET  12 |  | 39.06 | 0.00 | 0.00 |
| 13 | D:ALA  13 |  | 66.47 | 0.00 | 0.00 |
| 14 | D:LEU  14 |  | 25.30 | 0.00 | 0.00 |
| 15 | D:ILE  15 |  | 11.29 | 0.00 | 0.00 |
| 16 | D:GLN  16 | H | 21.51 | 17.49  \|\|\|\|\|\|\|\|\| | -0.21 |
| 17 | D:PRO  17 |  | 66.76 | 41.72  \|\|\|\|\|\|\| | 0.67 |
| 18 | D:GLY  18 |  | 5.04 | 1.19  \|\|\| | -0.01 |
| 19 | D:PHE  19 |  | 37.64 | 0.00 | 0.00 |
| 20 | D:SER  20 |  | 9.81 | 0.00 | 0.00 |
| 21 | D:LEU  21 |  | 15.21 | 0.00 | 0.00 |
| 22 | D:MET  22 |  | 0.17 | 0.00 | 0.00 |
| 23 | D:ASN  23 |  | 67.61 | 0.00 | 0.00 |
| 24 | D:PHE  24 |  | 15.38 | 0.00 | 0.00 |
| 25 | D:ASP  25 |  | 116.37 | 0.00 | 0.00 |
| 26 | D:GLY  26 |  | 59.92 | 0.00 | 0.00 |
| 27 | D:GLN  27 |  | 81.00 | 0.00 | 0.00 |
| 28 | D:ILE  28 |  | 7.90 | 0.00 | 0.00 |
| 29 | D:PHE  29 |  | 13.44 | 0.00 | 0.00 |
| 30 | D:PHE  30 |  | 12.99 | 0.00 | 0.00 |
| 31 | D:PHE  31 |  | 3.45 | 0.00 | 0.00 |
| 32 | D:GLY  32 |  | 4.46 | 0.00 | 0.00 |
| 33 | D:GLN  33 |  | 9.92 | 1.23  \|\| | -0.01 |
| 34 | D:LYS  34 | S | 137.25 | 66.60  \|\|\|\|\| | 0.33 |
| 35 | D:GLY  35 | H | 33.04 | 31.53  \|\|\|\|\|\|\|\|\|\| | 0.40 |
| 36 | D:TRP  36 |  | 142.31 | 79.30  \|\|\|\|\|\| | 0.69 |
| 37 | D:PRO  37 |  | 42.52 | 0.00 | 0.00 |
| 38 | D:LYS  38 |  | 139.44 | 0.00 | 0.00 |
| 39 | D:ARG  39 | HS | 231.94 | 74.96  \|\|\|\| | -0.23 |
| 40 | D:SER  40 |  | 94.90 | 0.00 | 0.00 |
| 41 | D:CYS  41 |  | 2.40 | 0.00 | 0.00 |
| 42 | D:PRO  42 |  | 81.86 | 3.18  \| | 0.05 |
| 43 | D:THR  43 |  | 10.30 | 0.00 | 0.00 |
| 44 | D:GLY  44 |  | 3.07 | 0.00 | 0.00 |
| 45 | D:VAL  45 |  | 2.67 | 0.00 | 0.00 |
| 46 | D:PHE  46 |  | 17.67 | 0.00 | 0.00 |
| 47 | D:HIS  47 |  | 29.37 | 0.00 | 0.00 |
| 48 | D:PHE  48 |  | 4.36 | 0.00 | 0.00 |
| 49 | D:ASP  49 |  | 36.21 | 0.00 | 0.00 |
| 50 | D:VAL  50 |  | 45.86 | 0.00 | 0.00 |
| 51 | D:LYS  51 |  | 84.38 | 0.00 | 0.00 |
| 52 | D:HIS  52 |  | 157.13 | 0.00 | 0.00 |
| 53 | D:ASN  53 |  | 83.81 | 0.00 | 0.00 |
| 54 | D:HIS  54 |  | 81.98 | 0.00 | 0.00 |
| 55 | D:LEU  55 |  | 5.54 | 0.00 | 0.00 |
| 56 | D:LYS  56 |  | 90.26 | 0.00 | 0.00 |
| 57 | D:LEU  57 |  | 19.30 | 0.00 | 0.00 |
| 58 | D:LYS  58 |  | 100.07 | 0.00 | 0.00 |
| 59 | D:PRO  59 |  | 69.64 | 0.00 | 0.00 |
| 60 | D:ALA  60 |  | 9.11 | 0.00 | 0.00 |
| 61 | D:LEU  61 |  | 94.53 | 0.00 | 0.00 |
| 62 | D:PHE  62 |  | 93.53 | 1.56  \| | 0.02 |
| 63 | D:SER  63 |  | 17.23 | 0.00 | 0.00 |
| 64 | D:LYS  64 |  | 203.79 | 0.00 | 0.00 |
| 65 | D:ASP  65 |  | 94.43 | 0.00 | 0.00 |
| 66 | D:SER  66 |  | 18.84 | 0.00 | 0.00 |
| 67 | D:CYS  67 |  | 69.03 | 0.00 | 0.00 |
| 68 | D:TYR  68 |  | 187.75 | 97.33  \|\|\|\|\|\| | 0.35 |
| 69 | D:LEU  69 |  | 16.60 | 0.00 | 0.00 |
| 70 | D:PRO  70 |  | 30.91 | 0.00 | 0.00 |
| 71 | D:PRO  71 |  | 2.74 | 0.70  \|\|\| | 0.00 |
| 72 | D:LEU  72 |  | 0.74 | 0.00 | 0.00 |
| 73 | D:ARG  73 | S | 39.16 | 32.32  \|\|\|\|\|\|\|\|\| | -0.77 |
| 74 | D:TYR  74 |  | 71.62 | 56.93  \|\|\|\|\|\|\|\| | 0.31 |
| 75 | D:PRO  75 |  | 17.83 | 0.00 | 0.00 |
| 76 | D:ALA  76 |  | 2.17 | 0.00 | 0.00 |
| 77 | D:THR  77 |  | 53.84 | 0.00 | 0.00 |
| 78 | D:CYS  78 |  | 6.30 | 0.00 | 0.00 |
| 79 | D:THR  79 |  | 72.76 | 0.00 | 0.00 |
| 80 | D:PHE  80 |  | 42.43 | 0.00 | 0.00 |
| 81 | D:LYS  81 |  | 117.22 | 0.00 | 0.00 |
| 82 | D:SER  82 |  | 8.47 | 0.00 | 0.00 |
| 83 | D:SER  83 |  | 76.01 | 0.00 | 0.00 |
| 84 | D:LEU  84 |  | 185.41 | 0.00 | 0.00 |
| 85 | D:GLU  85 |  | 134.22 | 0.00 | 0.00 |
| 86 | D:SER  86 |  | 51.00 | 0.00 | 0.00 |
| 87 | D:GLU  87 |  | 163.32 | 0.00 | 0.00 |
| 88 | D:LYS  88 |  | 88.07 | 0.00 | 0.00 |
| 89 | D:HIS  89 |  | 55.12 | 0.00 | 0.00 |
| 90 | D:GLN  90 |  | 48.88 | 0.00 | 0.00 |
| 91 | D:TYR  91 |  | 10.52 | 0.00 | 0.00 |
| 92 | D:ILE  92 |  | 0.50 | 0.00 | 0.00 |
| 93 | D:ILE  93 |  | 0.00 | 0.00 | 0.00 |
| 94 | D:HIS  94 |  | 2.90 | 0.00 | 0.00 |
| 95 | D:GLY  95 |  | 1.10 | 0.00 | 0.00 |
| 96 | D:GLY  96 |  | 0.00 | 0.00 | 0.00 |
| 97 | D:LYS  97 |  | 48.75 | 26.21  \|\|\|\|\|\| | -0.84 |
| 98 | D:THR  98 |  | 16.60 | 0.00 | 0.00 |
| 99 | D:PRO  99 | H | 25.47 | 24.67  \|\|\|\|\|\|\|\|\|\| | -0.10 |
| 100 | D:ASN 100 |  | 105.35 | 65.85  \|\|\|\|\|\|\| | -0.19 |
| 101 | D:ASN 101 |  | 55.80 | 37.91  \|\|\|\|\|\|\| | -0.15 |
| 102 | D:GLU 102 |  | 112.95 | 12.99  \|\| | -0.18 |
| 103 | D:LEU 103 |  | 34.07 | 0.00 | 0.00 |
| 104 | D:SER 104 |  | 21.55 | 0.00 | 0.00 |
| 105 | D:ASP 105 |  | 47.35 | 0.00 | 0.00 |
| 106 | D:LYS 106 |  | 105.78 | 0.00 | 0.00 |
| 107 | D:ILE 107 |  | 7.13 | 0.00 | 0.00 |
| 108 | D:TYR 108 |  | 11.57 | 0.00 | 0.00 |
| 109 | D:VAL 109 |  | 5.73 | 0.00 | 0.00 |
| 110 | D:MET 110 |  | 5.97 | 0.00 | 0.00 |
| 111 | D:SER 111 |  | 13.55 | 0.00 | 0.00 |
| 112 | D:VAL 112 |  | 41.94 | 0.00 | 0.00 |
| 113 | D:VAL 113 |  | 45.81 | 0.00 | 0.00 |
| 114 | D:CYS 114 |  | 70.13 | 0.00 | 0.00 |
| 115 | D:LYS 115 |  | 134.46 | 0.00 | 0.00 |
| 116 | D:ASN 116 |  | 149.81 | 0.00 | 0.00 |
| 117 | D:ASN 117 |  | 120.84 | 0.00 | 0.00 |
| 118 | D:LYS 118 |  | 199.21 | 0.00 | 0.00 |
| 119 | D:LYS 119 |  | 141.18 | 0.00 | 0.00 |
| 120 | D:VAL 120 |  | 42.34 | 0.00 | 0.00 |
| 121 | D:THR 121 |  | 27.00 | 0.00 | 0.00 |
| 122 | D:PHE 122 |  | 23.67 | 0.00 | 0.00 |
| 123 | D:ARG 123 |  | 83.89 | 0.00 | 0.00 |
| 124 | D:CYS 124 |  | 2.55 | 0.00 | 0.00 |
| 125 | D:ARG 125 |  | 151.70 | 0.00 | 0.00 |
| 126 | D:GLU 126 |  | 56.15 | 0.00 | 0.00 |
| 127 | D:LYS 127 |  | 26.04 | 0.00 | 0.00 |
| 128 | D:ASP 128 |  | 118.34 | 0.00 | 0.00 |
| 129 | D:LEU 129 |  | 39.13 | 0.00 | 0.00 |
| 130 | D:VAL 130 |  | 101.38 | 0.00 | 0.00 |
| 131 | D:GLY 131 |  | 47.40 | 0.00 | 0.00 |
| 132 | D:ASP 132 |  | 73.28 | 0.00 | 0.00 |
| 133 | D:VAL 133 |  | 60.62 | 0.00 | 0.00 |
| 134 | D:PRO 134 |  | 0.84 | 0.00 | 0.00 |
| 135 | D:GLU 135 |  | 116.30 | 0.00 | 0.00 |
| 136 | D:GLY 136 |  | 15.73 | 0.00 | 0.00 |
| 137 | D:ARG 137 |  | 8.75 | 0.00 | 0.00 |
| 138 | D:TYR 138 |  | 20.07 | 11.95  \|\|\|\|\|\| | 0.08 |
| 139 | D:GLY 139 |  | 9.45 | 2.30  \|\|\| | 0.03 |
| 140 | D:HIS 140 |  | 9.67 | 0.00 | 0.00 |
| 141 | D:SER 141 |  | 15.68 | 0.00 | 0.00 |
| 142 | D:ILE 142 |  | 6.81 | 0.00 | 0.00 |
| 143 | D:ASP 143 |  | 17.06 | 0.00 | 0.00 |
| 144 | D:VAL 144 |  | 18.76 | 0.00 | 0.00 |
| 145 | D:VAL 145 |  | 0.67 | 0.00 | 0.00 |
| 146 | D:TYR 146 |  | 65.28 | 0.00 | 0.00 |
| 147 | D:SER 147 |  | 6.90 | 0.00 | 0.00 |
| 148 | D:ARG 148 |  | 199.42 | 0.00 | 0.00 |
| 149 | D:GLY 149 |  | 64.42 | 0.00 | 0.00 |
| 150 | D:LYS 150 |  | 105.83 | 0.00 | 0.00 |
| 151 | D:SER 151 |  | 33.25 | 0.00 | 0.00 |
| 152 | D:MET 152 |  | 32.27 | 0.00 | 0.00 |
| 153 | D:GLY 153 |  | 0.48 | 0.00 | 0.00 |
| 154 | D:VAL 154 |  | 5.90 | 0.00 | 0.00 |
| 155 | D:LEU 155 |  | 1.84 | 0.00 | 0.00 |
| 156 | D:PHE 156 |  | 4.05 | 0.00 | 0.00 |
| 157 | D:GLY 157 |  | 7.69 | 0.00 | 0.00 |
| 158 | D:GLY 158 |  | 0.61 | 0.00 | 0.00 |
| 159 | D:ARG 159 | HS | 39.43 | 38.70  \|\|\|\|\|\|\|\|\|\| | -1.09 |
| 160 | D:SER 160 |  | 18.29 | 0.00 | 0.00 |
| 161 | D:TYR 161 |  | 40.79 | 0.40  \| | -0.00 |
| 162 | D:ILE 162 |  | 26.71 | 0.00 | 0.00 |
| 163 | D:PRO 163 |  | 90.33 | 0.00 | 0.00 |
| 164 | D:SER 164 |  | 113.19 | 11.66  \|\| | -0.13 |
| 165 | D:ALA 165 |  | 90.11 | 0.12  \| | -0.00 |
| 166 | D:GLN 166 |  | 107.31 | 0.00 | 0.00 |
| 167 | D:ARG 167 |  | 66.98 | 24.31  \|\|\|\| | -0.10 |
| 168 | D:THR 168 |  | 60.93 | 35.87  \|\|\|\|\|\| | 0.55 |
| 169 | D:THR 169 | H | 133.81 | 131.36  \|\|\|\|\|\|\|\|\|\| | 0.66 |
| 170 | D:GLU 170 | S | 168.34 | 136.87  \|\|\|\|\|\|\|\|\| | -0.25 |
| 171 | D:LYS 171 |  | 86.44 | 0.97  \| | 0.02 |
| 172 | D:TRP 172 | H | 114.96 | 66.12  \|\|\|\|\|\| | 0.63 |
| 173 | D:ASN 173 | H | 110.83 | 109.94  \|\|\|\|\|\|\|\|\|\| | -0.31 |
| 174 | D:SER 174 | H | 56.70 | 22.39  \|\|\|\| | 0.29 |
| 175 | D:VAL 175 | H | 48.34 | 22.96  \|\|\|\|\| | 0.34 |
| 176 | D:ALA 176 |  | 45.24 | 0.00 | 0.00 |
| 177 | D:ASP 177 |  | 31.80 | 0.00 | 0.00 |
| 178 | D:CYS 178 |  | 19.60 | 0.00 | 0.00 |
| 179 | D:LEU 179 |  | 142.41 | 0.00 | 0.00 |
| 180 | D:PRO 180 |  | 19.27 | 0.00 | 0.00 |
| 181 | D:HIS 181 |  | 56.08 | 0.00 | 0.00 |
| 182 | D:ILE 182 |  | 1.84 | 0.00 | 0.00 |
| 183 | D:PHE 183 |  | 16.00 | 0.00 | 0.00 |
| 184 | D:LEU 184 |  | 6.06 | 0.00 | 0.00 |
| 185 | D:VAL 185 |  | 3.86 | 0.00 | 0.00 |
| 186 | D:ASP 186 |  | 33.90 | 0.00 | 0.00 |
| 187 | D:PHE 187 |  | 28.84 | 0.00 | 0.00 |
| 188 | D:GLU 188 |  | 126.61 | 0.00 | 0.00 |
| 189 | D:PHE 189 |  | 151.94 | 0.00 | 0.00 |
| 190 | D:GLY 190 |  | 13.08 | 0.00 | 0.00 |
| 191 | D:CYS 191 |  | 64.01 | 0.00 | 0.00 |
| 192 | D:SER 192 |  | 5.65 | 0.00 | 0.00 |
| 193 | D:THR 193 |  | 50.45 | 0.00 | 0.00 |
| 194 | D:SER 194 |  | 38.59 | 0.00 | 0.00 |
| 195 | D:TYR 195 |  | 50.00 | 0.00 | 0.00 |
| 196 | D:ILE 196 |  | 119.01 | 0.00 | 0.00 |
| 197 | D:LEU 197 |  | 16.84 | 0.00 | 0.00 |
| 198 | D:PRO 198 |  | 114.39 | 0.00 | 0.00 |
| 199 | D:GLU 199 |  | 76.51 | 0.00 | 0.00 |
| 200 | D:LEU 200 |  | 9.40 | 0.00 | 0.00 |
| 201 | D:GLN 201 |  | 159.20 | 0.00 | 0.00 |
| 202 | D:ASP 202 |  | 67.65 | 0.00 | 0.00 |
| 203 | D:GLY 203 |  | 2.24 | 0.00 | 0.00 |
| 204 | D:LEU 204 |  | 9.98 | 0.00 | 0.00 |
| 205 | D:SER 205 |  | 0.00 | 0.00 | 0.00 |
| 206 | D:PHE 206 |  | 44.29 | 43.51  \|\|\|\|\|\|\|\|\|\| | 0.70 |
| 207 | D:HIS 207 |  | 15.03 | 0.12  \| | -0.00 |
| 208 | D:VAL 208 |  | 14.74 | 0.00 | 0.00 |
| 209 | D:SER 209 |  | 18.95 | 0.00 | 0.00 |
| 210 | D:ILE 210 |  | 4.52 | 0.00 | 0.00 |
| 211 | D:ALA 211 |  | 30.66 | 0.00 | 0.00 |
| 212 | D:ARG 212 |  | 62.34 | 0.00 | 0.00 |
| 213 | D:ASN 213 |  | 103.74 | 0.00 | 0.00 |
| 214 | D:ASP 214 |  | 24.24 | 0.00 | 0.00 |
| 215 | D:THR 215 |  | 23.37 | 0.00 | 0.00 |
| 216 | D:ILE 216 |  | 2.51 | 0.00 | 0.00 |
| 217 | D:TYR 217 |  | 5.68 | 0.00 | 0.00 |
| 218 | D:ILE 218 |  | 3.17 | 0.00 | 0.00 |
| 219 | D:LEU 219 |  | 1.81 | 0.00 | 0.00 |
| 220 | D:GLY 220 |  | 0.46 | 0.00 | 0.00 |
| 221 | D:GLY 221 |  | 1.41 | 0.00 | 0.00 |
| 222 | D:HIS 222 |  | 24.13 | 5.11  \|\|\| | -0.06 |
| 223 | D:SER 223 |  | 10.96 | 0.00 | 0.00 |
| 224 | D:LEU 224 |  | 51.28 | 2.01  \| | 0.03 |
| 225 | D:ALA 225 |  | 83.44 | 0.00 | 0.00 |
| 226 | D:ASN 226 |  | 105.01 | 0.00 | 0.00 |
| 227 | D:ASN 227 |  | 109.13 | 0.00 | 0.00 |
| 228 | D:ILE 228 |  | 89.94 | 0.00 | 0.00 |
| 229 | D:ARG 229 |  | 46.92 | 5.24  \|\| | -0.19 |
| 230 | D:PRO 230 |  | 27.25 | 0.00 | 0.00 |
| 231 | D:ALA 231 |  | 53.97 | 0.00 | 0.00 |
| 232 | D:ASN 232 |  | 66.49 | 0.00 | 0.00 |
| 233 | D:LEU 233 |  | 0.33 | 0.00 | 0.00 |
| 234 | D:TYR 234 |  | 43.12 | 0.00 | 0.00 |
| 235 | D:LYS 235 |  | 38.60 | 0.00 | 0.00 |
| 236 | D:ILE 236 |  | 1.64 | 0.00 | 0.00 |
| 237 | D:ARG 237 |  | 165.40 | 0.00 | 0.00 |
| 238 | D:VAL 238 |  | 1.17 | 0.00 | 0.00 |
| 239 | D:ASP 239 |  | 45.92 | 0.00 | 0.00 |
| 240 | D:LEU 240 |  | 15.46 | 0.00 | 0.00 |
| 241 | D:PRO 241 |  | 51.27 | 0.00 | 0.00 |
| 242 | D:LEU 242 |  | 174.42 | 0.00 | 0.00 |
| 243 | D:GLY 243 |  | 66.56 | 0.00 | 0.00 |
| 244 | D:SER 244 |  | 75.33 | 0.00 | 0.00 |
| 245 | D:PRO 245 |  | 23.97 | 0.00 | 0.00 |
| 246 | D:ALA 246 |  | 68.94 | 0.00 | 0.00 |
| 247 | D:VAL 247 |  | 33.63 | 0.00 | 0.00 |
| 248 | D:THR 248 |  | 87.74 | 0.00 | 0.00 |
| 249 | D:CYS 249 |  | 30.84 | 0.00 | 0.00 |
| 250 | D:THR 250 |  | 54.33 | 0.00 | 0.00 |
| 251 | D:VAL 251 |  | 68.16 | 0.00 | 0.00 |
| 252 | D:LEU 252 |  | 32.63 | 0.00 | 0.00 |
| 253 | D:PRO 253 |  | 124.65 | 0.00 | 0.00 |
| 254 | D:GLY 254 |  | 51.07 | 0.00 | 0.00 |
| 255 | D:GLY 255 |  | 21.47 | 0.00 | 0.00 |
| 256 | D:ILE 256 |  | 14.72 | 0.00 | 0.00 |
| 257 | D:SER 257 |  | 34.48 | 0.00 | 0.00 |
| 258 | D:VAL 258 |  | 0.00 | 0.00 | 0.00 |
| 259 | D:SER 259 |  | 10.56 | 7.54  \|\|\|\|\|\|\|\| | 0.02 |
| 260 | D:SER 260 | H | 58.79 | 38.66  \|\|\|\|\|\|\| | 0.31 |
| 261 | D:ALA 261 |  | 24.48 | 0.00 | 0.00 |
| 262 | D:ILE 262 |  | 13.03 | 0.00 | 0.00 |
| 263 | D:LEU 263 |  | 38.40 | 0.00 | 0.00 |
| 264 | D:THR 264 |  | 2.83 | 0.00 | 0.00 |
| 265 | D:GLN 265 |  | 56.68 | 0.00 | 0.00 |
| 266 | D:THR 266 |  | 47.53 | 0.00 | 0.00 |
| 267 | D:SER 267 |  | 62.97 | 0.00 | 0.00 |
| 268 | D:SER 268 |  | 91.92 | 0.00 | 0.00 |
| 269 | D:ASP 269 |  | 12.32 | 0.00 | 0.00 |
| 270 | D:GLU 270 |  | 42.52 | 0.00 | 0.00 |
| 271 | D:PHE 271 |  | 33.93 | 0.00 | 0.00 |
| 272 | D:VAL 272 |  | 2.68 | 0.00 | 0.00 |
| 273 | D:ILE 273 |  | 1.25 | 0.00 | 0.00 |
| 274 | D:VAL 274 |  | 1.11 | 0.00 | 0.00 |
| 275 | D:GLY 275 |  | 3.68 | 0.00 | 0.00 |
| 276 | D:GLY 276 |  | 1.84 | 0.00 | 0.00 |
| 277 | D:TYR 277 |  | 45.92 | 34.27  \|\|\|\|\|\|\|\| | 0.52 |
| 278 | D:GLN 278 |  | 56.93 | 0.00 | 0.00 |
| 279 | D:LEU 279 |  | 104.40 | 0.00 | 0.00 |
| 280 | D:GLU 280 |  | 109.42 | 0.00 | 0.00 |
| 281 | D:ASN 281 |  | 108.80 | 0.00 | 0.00 |
| 282 | D:GLN 282 |  | 95.20 | 0.00 | 0.00 |
| 283 | D:LYS 283 |  | 11.03 | 0.00 | 0.00 |
| 284 | D:ARG 284 |  | 27.49 | 0.00 | 0.00 |
| 285 | D:MET 285 |  | 37.45 | 0.00 | 0.00 |
| 286 | D:VAL 286 |  | 69.14 | 0.00 | 0.00 |
| 287 | D:CYS 287 |  | 2.80 | 0.00 | 0.00 |
| 288 | D:ASN 288 |  | 20.43 | 0.00 | 0.00 |
| 289 | D:ILE 289 |  | 33.51 | 0.00 | 0.00 |
| 290 | D:ILE 290 |  | 0.33 | 0.00 | 0.00 |
| 291 | D:SER 291 |  | 28.98 | 0.00 | 0.00 |
| 292 | D:PHE 292 |  | 5.23 | 0.00 | 0.00 |
| 293 | D:LYS 293 |  | 111.05 | 0.00 | 0.00 |
| 294 | D:ASP 294 |  | 128.30 | 0.00 | 0.00 |
| 295 | D:ASN 295 |  | 136.40 | 0.00 | 0.00 |
| 296 | D:LYS 296 |  | 109.63 | 0.00 | 0.00 |
| 297 | D:ILE 297 |  | 15.24 | 0.00 | 0.00 |
| 298 | D:GLY 298 |  | 24.46 | 0.00 | 0.00 |
| 299 | D:ILE 299 |  | 52.68 | 0.00 | 0.00 |
| 300 | D:HIS 300 |  | 127.51 | 0.00 | 0.00 |
| 301 | D:GLU 301 |  | 145.62 | 0.00 | 0.00 |
| 302 | D:MET 302 |  | 68.86 | 0.00 | 0.00 |
| 303 | D:GLU 303 |  | 154.42 | 0.00 | 0.00 |
| 304 | D:THR 304 |  | 37.16 | 0.00 | 0.00 |
| 305 | D:PRO 305 |  | 11.18 | 0.00 | 0.00 |
| 306 | D:ASP 306 |  | 134.68 | 0.00 | 0.00 |
| 307 | D:TRP 307 |  | 23.60 | 0.00 | 0.00 |
| 308 | D:THR 308 |  | 45.74 | 0.00 | 0.00 |
| 309 | D:PRO 309 |  | 96.30 | 0.00 | 0.00 |
| 310 | D:ASP 310 |  | 97.90 | 0.00 | 0.00 |
| 311 | D:ILE 311 |  | 0.00 | 0.00 | 0.00 |
| 312 | D:LYS 312 |  | 116.39 | 0.00 | 0.00 |
| 313 | D:HIS 313 |  | 147.55 | 0.00 | 0.00 |
| 314 | D:SER 314 |  | 20.14 | 0.00 | 0.00 |
| 315 | D:LYS 315 |  | 130.12 | 7.20  \| | 0.12 |
| 316 | D:ILE 316 |  | 76.36 | 10.04  \|\| | 0.16 |
| 317 | D:TRP 317 |  | 13.49 | 4.05  \|\|\|\| | -0.05 |
| 318 | D:PHE 318 |  | 27.19 | 0.50  \| | 0.01 |
| 319 | D:GLY 319 |  | 19.85 | 0.00 | 0.00 |
| 320 | D:SER 320 |  | 21.46 | 0.00 | 0.00 |
| 321 | D:ASN 321 |  | 27.16 | 0.00 | 0.00 |
| 322 | D:MET 322 |  | 38.39 | 0.00 | 0.00 |
| 323 | D:GLY 323 |  | 7.24 | 0.00 | 0.00 |
| 324 | D:ASN 324 |  | 130.62 | 0.00 | 0.00 |
| 325 | D:GLY 325 |  | 5.10 | 0.00 | 0.00 |
| 326 | D:THR 326 |  | 7.50 | 0.00 | 0.00 |
| 327 | D:VAL 327 |  | 2.01 | 0.00 | 0.00 |
| 328 | D:PHE 328 |  | 13.28 | 0.00 | 0.00 |
| 329 | D:LEU 329 |  | 3.85 | 0.00 | 0.00 |
| 330 | D:GLY 330 |  | 2.59 | 0.00 | 0.00 |
| 331 | D:ILE 331 |  | 8.54 | 0.00 | 0.00 |
| 332 | D:PRO 332 |  | 48.10 | 15.08  \|\|\|\| | 0.22 |
| 333 | D:GLY 333 |  | 26.15 | 3.07  \|\| | -0.04 |
| 334 | D:ASP 334 |  | 144.12 | 56.69  \|\|\|\| | -0.42 |
| 335 | D:ASN 335 |  | 98.20 | 10.45  \|\| | -0.11 |
| 336 | D:LYS 336 |  | 182.80 | 74.71  \|\|\|\|\| | -0.32 |
| 337 | D:GLN 337 |  | 141.84 | 23.57  \|\| | -0.29 |
| 338 | D:ALA 338 |  | 77.98 | 9.40  \|\| | 0.04 |
| 339 | D:LEU 339 |  | 79.99 | 0.00 | 0.00 |
| 340 | D:SER 340 |  | 80.11 | 0.00 | 0.00 |
| 341 | D:GLU 341 |  | 38.21 | 0.00 | 0.00 |
| 342 | D:ALA 342 |  | 44.17 | 0.00 | 0.00 |
| 343 | D:PHE 343 |  | 24.31 | 0.00 | 0.00 |
| 344 | D:TYR 344 |  | 96.24 | 0.00 | 0.00 |
| 345 | D:PHE 345 |  | 12.83 | 0.00 | 0.00 |
| 346 | D:TYR 346 |  | 13.10 | 0.00 | 0.00 |
| 347 | D:THR 347 |  | 17.86 | 0.00 | 0.00 |
| 348 | D:LEU 348 |  | 0.77 | 0.00 | 0.00 |
| 349 | D:LYS 349 |  | 104.81 | 0.00 | 0.00 |
| 350 | D:CYS 350 |  | 24.75 | 0.00 | 0.00 |
| 351 | D:THR 351 |  | 154.87 | 0.00 | 0.00 |

**Supplementary Table S4.** Interfacing residues of RAG2 in RAG1-RAG2.

| Site ID | Primer sequence (5’–3’) |
| --- | --- |
| RAG1-sgRNA | Forward: CTTGTCCTTATTGCTCCCAGGT  Reverse: AGATTTCACAAAGTGCGCCG |
| RAG2-sgRNA | Forward: GTTCTCTTTGGAGGACGGTCA  Reverse: ATCACAAGTAGGGCAGCATGT |
| RAG1-PCR | Forward: CTTGTCCTTATTGCTCCCAGGT  Reverse: AGATTTCACAAAGTGCGCCG |
| RAG2-PCR | Forward: GTTCTCTTTGGAGGACGGTCA  Reverse: ATCACAAGTAGGGCAGCATGT |
| D1J1 | Forward: GCTGCTCTGGTGGTTTCTCAC  Reverse: CACCAGTGCCCAAGTCTTAGC |
| D4-J3 | Forward: CTGAGAACTCACGTCCAGTGC  Reverse: CTGGCCCTAGACCTTTAGACC |
| FR1-JH | Forward: GAGGAGAAGCTGGTGGAGT  Reverse: TGAGGACACGACGACTTCAA |
